# Association of Urinary Collagen Type III Degradation Product With Kidney Function and Fibrosis in Chronic Kidney Disease Patients

**DOI:** 10.1002/pmic.202400354

**Published:** 2025-04-10

**Authors:** Emily M. Martin, Federica Genovese, Harald Mischak, Justyna Siwy, Harald Rupprecht, Lorenzo Catanese, Agnieszka Latosinska

**Affiliations:** ^1^ Nordic Bioscience A/S Herlev Denmark; ^2^ University of Copenhagen Copenhagen Denmark; ^3^ Mosaiques Diagnostics GmbH Hannover Germany; ^4^ Department of Nephrology, Angiology and Rheumatology Klinikum Bayreuth GmbH Bayreuth Germany; ^5^ Department of Nephrology Medizincampus Oberfranken Friedrich‐Alexander‐University Erlangen‐Nürnberg Erlangen Germany; ^6^ Kuratorium for Dialysis and Transplantation (KfH), Bayreuth Bayreuth Germany

**Keywords:** chronic kidney disease, collagen type III, fibrosis, peptides, urine

## Abstract

A common hallmark of chronic kidney disease (CKD) is kidney fibrosis, which manifests as an increased deposition and turnover of collagens in the kidneys. Current clinical methods of monitoring disease progression in CKD patients do not truly reflect alterations at the tissue level without the use of invasive biopsies. Naturally occurring urinary peptides associated with kidney function and fibrosis have been previously identified using CE‐MS as a non‐invasive alternative. Moreover, a specific peptide from collagen type III, a highly abundant interstitial collagen, is the target for the C3M enzyme‐linked immunosorbent assay (ELISA)‐based assay and has been recorded in the CKD273 urinary biomarker panel measured by CE‐MS. We aimed to investigate the intensities of the peptides incorporating the C3M sequence captured by CE‐MS in urine of patients with CKD and analyze their association with estimated glomerular filtration rate (eGFR) and kidney interstitial fibrosis and tubular atrophy (IFTA). The investigated collagen type III peptides were reduced in abundance in urine of patients with CKD compared to healthy controls and the peptide intensities were independently correlated to eGFR and inversely correlated with IFTA score. Collectively, this analysis supports that peptides containing the C3M sequence are significantly associated with kidney function decline and tissue fibrosis.

AbbreviationsCE‐MScapillary electrophoresis coupled to mass spectrometryCKDchronic kidney diseaseeGFRestimated glomerular filtration rateIFTAinterstitial fibrosis and tubular atrophy

## Introduction

1

**FIGURE 1 pmic13953-fig-0001:**
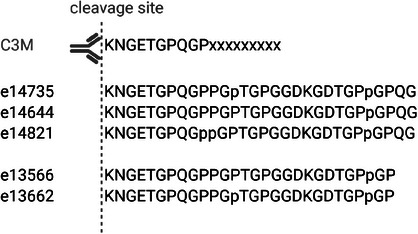
*Alignment of the C3*
*M target sequence with collagen type III‐derived peptides from CE‐MS used in this analysis. The C3*
*M target sequence could include amino acids after the 10 amino acid target, marked by ‘xxx’, but this does not impact the C3*
*M assay. Made in biorender.com*.

Kidney fibrosis is a common consequence of kidney injury due to different causes, and it often results in kidney function decline and, ultimately, kidney failure. While there is a relationship between estimated glomerular filtration rate (eGFR) decline and increasing interstitial fibrosis and tubular atrophy (IFTA), structural tissue changes happen before kidney function decline is detectable; hence, methods to monitor kidney fibrosis without the use of invasive biopsies are sought [1]. A possible non‐invasive approach is the use of urinary collagen biomarkers that reflect the dynamics of the kidney connective tissue turnover [2, 3, 4]. The kidney fibrotic scar is primarily constituted of type I and type III collagen deposits in the tubulointerstitial space [5]. The homotrimeric collagen type III, encoded by *COL3A1*, is expressed at low levels in the interstitium and at negligible levels in the glomerulus in physiological conditions; however, in fibrotic conditions, there is an upregulation of COL3A1 and deposition of collagen type III in the interstitium and glomeruli [2, 6, 7]. Collagen type III turnover has been attributed to an early‐stage chronic kidney disease (CKD) phenotype in cluster analyses, indicating early collagen type III changes in disease [8]. C3M is a biomarker measured with the nordicC3M enzyme‐linked immunosorbent assay (ELISA), targeting the sequence ^610^KNGETGPQGP^619^ (specifically the cleaved N‐terminal end of the sequence) (Figure 1) [9]. Urinary C3M (uC3M) has been positively associated with eGFR in multiple CKD indications [10, 11, 12, 13] and has been shown to decrease with an increase in CKD stage [10, 14]. Low levels of uC3M at baseline were associated with an increased risk of CKD progression [14], and development of end‐stage kidney disease [10]. uC3M was strongly and inversely correlated with interstitial fibrosis (%, Banff and T‐score) [11], tubular atrophy (%), and interstitial mononuclear cell infiltration [13] in kidney biopsy collected at the same time of sampling. On the contrary, serum levels of C3M did not change across different CKD stages [14]. C3M in serum reflects an inflammatory phenotype, as shown by strong associations with inflammation markers [10, 14, 15]. The sequence recognized by the C3M antibody has been found in several peptides contained in urine of CKD patients detected by capillary electrophoresis coupled to mass spectrometry (CE‐MS), where some of these are part of the CKD273 classifier [16, 17]. The peptide ^610^KNGETGPQGPPGPTGPGGDKGDTGPPGPQG^639^ was experimentally found to be cleaved specifically by matrix metalloproteinase‐9 (MMP‐9) [18]. It was correlated with eGFR among those included in CKD273 classifier [16, 19] and has been previously shown to be reduced in patients with CKD compared to controls [17, 19]. This peptide abundance is also affected in diabetic patients [20]. Here, we conducted a study investigating the association of the collagen type III‐derived urinary peptides with eGFR and IFTA using CE‐MS. Specifically, we assessed the extent of correlation of the peptides encompassing the C3M sequence to kidney function decline (measured by eGFR), and IFTA score in a large number of data points.

Previously acquired data were used to explore these correlations: a CKD case‐control study [21] and a CKD cohort with IFTA score recorded [22] (Table S1). The CKD case‐control study includes 612 patients with CKD and 612 non‐CKD controls, with matched sex and age (62% male, 60 years), an eGFR of 49 mL/min/1.73m^2^ in the CKD group versus 97 mL/min/1.73m^2^ in the controls. In the CKD cohort with IFTA score (*n* = 466), patients were predominantly male (59%) with an average age of 58 and had an eGFR of 30 mL/min/1.73m^2^ with 15% IFTA score. CE‐MS anonymized datasets were extracted from the Human Urinary Proteome Database [23]. The study was conducted according to the guidelines of the Declaration of Helsinki. All datasets were fully anonymized. The ethics committee of the Hannover Medical School Germany waived ethical approval under reference number 3116‐2016 for all studies involving reuse of data from anonymized urine samples. Sample analysis and data processing have been described before [24]. The CE‐MS peptides had been normalized using a predefined set of 29 commonly occurring urinary peptides, using methods reported previously [24]. The five naturally occurring collagen type III peptides with the N‐terminus of the sequence targeted by the C3M assay that were detected using CE‐MS (e14735, e14644, e14821, e13566, e13662), have been assessed in this study. Intensities of these peptides are available in Table S2. These CE‐MS detected peptides differ in number of hydroxyproline and in sequence length (Figure 1). Statistical analysis was completed in RStudio (version 4.3.3) using the cor.test() and wilcox.test() functions for Spearman's rank correlation and Mann–Whitney *U*‐test, respectively. Data was log‐transformed to improve distribution but remained not normally distributed. Peptide intensities recorded as zero were excluded from the analysis unless otherwise stated.

Among the collagen type III peptides in the CE‐MS datasets, the e14375 peptide was observed with the highest frequency across all datasets (average 95% frequency), and the other peptides descended in frequency by e13566, e14644, e13662 to e14821 (73%, 52%, 50%, and 43% respectively) (Table S3).

**FIGURE 2 pmic13953-fig-0002:**
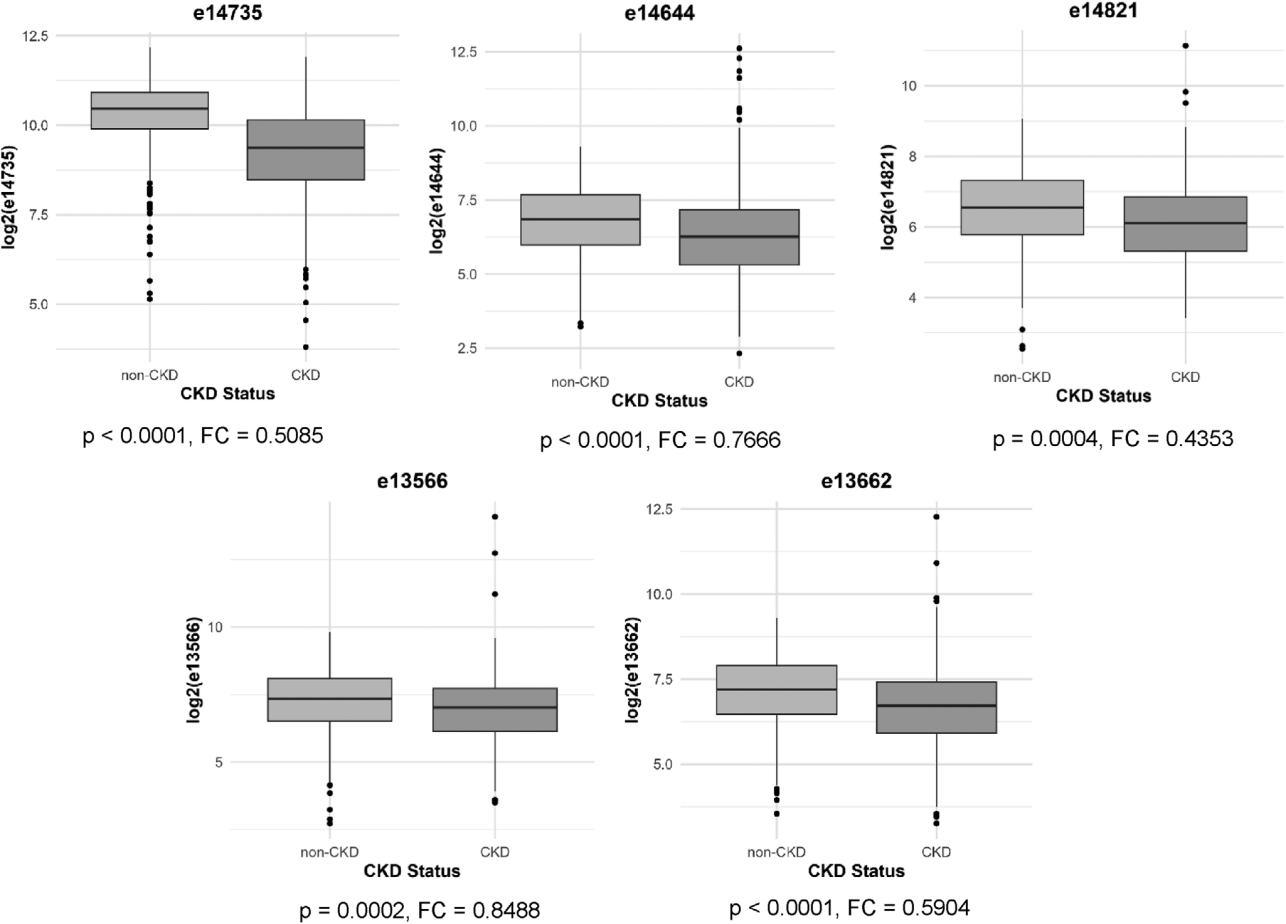
CKD individuals have decreased individual peptide intensities compared to non‐CKD controls. The intensities of the peptides (e14735, e13662, e13566, e14644, e14821) were log‐transformed and a Mann–Whitney *U*‐test was performed to compare CKD with non‐CKD individuals. A FC of the peptide intensity from CKD to non‐CKD individuals was calculated as a fraction of the mean of each group from linear (normalized) data. Peptide intensities marked as zero were retained in this analysis. CKD, chronic kidney disease; FC, fold change.

First, we wanted to confirm that the change in peptide intensity between a CKD group (eGFR <60 mL/min/1.73m^2^) and non‐CKD control group (eGFR>90 mL/min/1.73m^2^) decreased as previously shown for uC3M [11]. We observed that all five peptides were significantly reduced in the CKD group compared to the non‐CKD control group, with the fold change of the normalized peptide intensities ranging from 0.44 to 0.85 (Figure 2). Based on these results, peptide intensity was reduced by 15–56% in CKD individuals compared to the non‐CKD controls, supporting reduced excretion of the peptide in kidney pathology.

**FIGURE 3 pmic13953-fig-0003:**
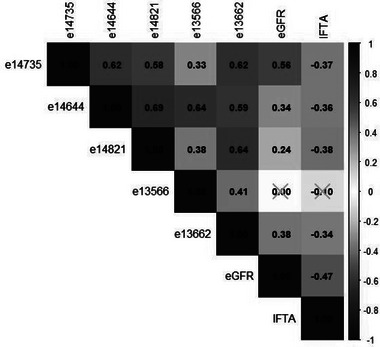
Individual peptide intensities are correlated to eGFR in the CKD cohort with IFTA score recorded. Most of the individual peptides were also highly and inversely correlated with IFTA (%) score. Spearman's rank correlations were performed on log‐transformed data and all correlations marked with a grey “*X*” were non‐significant correlations. For all peptide‐to‐peptide correlations, *p <* *0.0001*. Correlations to eGFR were: e14735, *p* < 0.0001; e14644, *p* < 0.0001; e14821, *p* = 0.0147; e13662, *p* < 0.0001, and correlations to IFTA were: e14735, *p* < 0.0001; e14644, *p* < 0.0001; e14821, *p* = 0.0005; e13662, *p* < 0.0001; eGFR, *p* < 0.0001. eGFR, estimated glomerular filtration rate; IFTA, interstitial fibrosis and tubular atrophy.

In a study with available biopsies including individuals with moderate to severe CKD, we observed that four out of five peptides were significantly correlated to eGFR (coefficient ranging 0.24–0.56) and inversely correlated to IFTA (%) score (ranging −0.34 to −0.38) (Figure 3), established on biopsies stained with Masson Trichome [4]. This association confirms the association of peptides containing uC3M targeted sequence to eGFR, particularly in cohorts with broader eGFR ranges [10, 11], and to fibrosis score observed in different biopsy studies [11, 25]. All CE‐MS measured peptides were highly significantly correlated with each other (Figure 3).

This study confirms that peptides containing the sequence detected by the uC3M assay may be relevant in CKD. However, it cannot be assumed that the peptides identified by the uC3M assay are specific to the kidney (similar applies for peptides detected by CE‐MS), even though differential levels of the sequence targeted by C3M have been measured in sera [14], suggesting a potentially more relevant role in urine and the kidney. Among the cohorts examined in this investigation, no cohort included uC3M measured with ELISA making it difficult to directly verify the previous findings with uC3M.

To conclude, this analysis supports that peptides containing the sequence measured by uC3 M ELISA assay are correlated to kidney function decline and fibrosis.

## Author contributions

Emily M. Martin completed the statistical analysis and wrote the initial version of the manuscript. Lorenzo Catanese and Harald Rupprecht provided the samples and clinical data. Agnieszka Latosinska, Justyna Siwy, and Harald Mischak performed the CE‐MS, and along with Federica Genovese provided critical input on the direction of the study and statistical analysis. All authors read and edited various drafts of the manuscript and approved the final submission.

## Conflicts of Interest

E.M.M. is an employee of Nordic Bioscience A/S as a Ph.D. student under the DisCo‐I Marie Skłodowska‐Curie Actions Doctoral Network. F.G. is an employee and shareholder of Nordic Bioscience A/S. H.M. is the founder and co‐owner of Mosaiques Diagnostics GmbH (Hannover, Germany). A.L. and J.S. are employed by Mosaiques Diagnostics GmbH.

## Consent

Informed consent given by study participants did not include data sharing with third parties. However, anonymized data can be made available to investigators for targeted non‐commercial research based on a motivated request to be submitted to EMM and pending ethical clearance by the Ethics Review Board of each institution involved in the study.

## References

## Supporting information



Supporting Information

Supporting Information

Supporting Information

## Data Availability

All relevant data are available in the article's supplementary material.
