# Supplementary material for: Association of Urinary Collagen Type III Degradation Product With Kidney Function and Fibrosis in Chronic Kidney Disease Patients
Source: Proteomics. 2025 Apr 10;25(11-12):e202400354. doi: 10.1002/pmic.202400354 (PMC12205290; doi:10.1002/pmic.202400354)
Supplement: Supplementary file 1 — Supporting Information [file PMIC-25-e202400354-s003.docx]

**Supplementary tables:**

**Table S1:** Basic clinical characteristics of each cohort used.

|  | **Case-control** | **IFTA dataset** |
| --- | --- | --- |
| **Study size** (n) | 1224 (612 CKD, 612 non-CKD) | 466 |
| **Age** (mean, years) | 60 | 58 |
| **Sex** (%, male) | 62 | 59 |
| **eGFR (ml/min/1.73m^2^)** (median [IQR]) | CKD cases: 49.1 [37.5 - 56.0]  Non-CKD controls: 97.0 [93.2 - 103.5] | 29.5 [13.3 - 52.6] |
| **IFTA (%)** (median [IQR]) | *NA* | 15.0 [5.0 - 30.0] |

*eGFR, estimated glomerular filtration rate; IFTA, interstitial fibrosis and tubular atrophy; CKD, chronic kidney disease; NA, not available.*

**Table S2:** Normalized peptide intensities per urine sample (analysis ID) in the case-control study (**A**) and the dataset with IFTA recorded (**B**).

**Table S3:** Frequency of peptides measured using CE-MS.

|  | **Case-control*** | **IFTA dataset** |
| --- | --- | --- |
| e14735 | 1176/1224 (96%) | 414/466 (89%) |
| e13566 | 841/1224 (69%) | 338/466 (72%) |
| e13662 | 615/1224 (50%) | 179/466 (38%) |
| e14644 | 658/1224 (54%) | 162/466 (35%) |
| e14821 | 527/1224 (43%) | 106/466 (23%) |

********for the case-control analysis, the missing peptide intensity data were replaced with zero and included in the analysis. IFTA, interstitial fibrosis and tubular atrophy.*
