# Supplementary material for: Association of Urinary Collagen Type III Degradation Product With Kidney Function and Fibrosis in Chronic Kidney Disease Patients
Source: Proteomics. 2025 Apr 10;25(11-12):e202400354. doi: 10.1002/pmic.202400354 (PMC12205290; doi:10.1002/pmic.202400354)
Supplement: Supplementary file 2 — Supporting Information [file PMIC-25-e202400354-s002.pdf]

Table S2A: Normalized peptide intensities per urine sample (analysis ID) in the case-control study.

Analysis ID	Group (0-control, 1-CKD)	e13566	e13662	e14644	e14735	e14821
120025	0	0	127.01	91.18	2380.1	101.04
122293	0	0	0	0	654.84	0
123595	0	0	0	0	654.2	0
123759	0	160.69	0	0	626.76	0
124148	0	117.62	0	84.23	682.8	0
124732	0	0	0	0	1445.42	0
125122	0	0	0	0	118.26	0
126036	0	53.93	39.2	0	619.97	41.23
127087	0	123.53	0	0	0	0
127112	0	0	0	0	50.35	0
127461	0	95.84	142.75	0	754.51	29.48
127465	0	167.71	0	352.02	1636.61	71.24
127493	0	230.17	0	144.37	1097.7	48.93
127503	0	0	0	352.01	2900.77	0
127518	0	0	0	0	459.25	0
127530	0	0	0	0	0	0
127545	0	44.69	0	10.15	214.51	0
127547	0	154.35	57.51	81.16	1631.6	0
128390	0	374.1	0	142.19	1582.05	0
128584	0	0	0	0	414.76	0
128729	0	0	0	92.62	1302.61	0
129325	0	81.87	0	43.95	442.22	26.16
129339	0	0	0	132.87	1617.03	122.79
129353	0	132.37	63.07	0	1044.32	70.58
129355	0	318.54	176.93	257.08	1519.43	73.67
129363	0	276.08	239.4	366.99	2920.57	264.69
129365	0	161.43	0	134.38	3223.55	378.64
129375	0	209.18	187.08	0	1245.55	0
129379	0	118.31	91.2	0	969.14	65.03
129384	0	304.11	337.75	285.1	1921.18	140.25
129386	0	191.57	263.1	260.84	2235.53	113.48
129390	0	45.32	171.46	80.14	1750.09	93.93
129394	0	167.28	97.02	192.87	2522.17	134.1
129396	0	307.08	225.69	245.79	2205.41	326.09
129398	0	303.93	0	183.35	1301.94	0
129402	0	0	0	58.16	1332.25	0
129413	0	394.04	386.58	314.85	3216.92	343.93
129415	0	494.53	309.21	192.65	3075.98	189.33
129423	0	229.35	140.81	62.15	963.95	61.93
129425	0	187.01	194.49	262.84	2725.54	336.23
129429	0	144.68	0	155.78	1688.09	61.95
129452	0	330.18	0	158.33	1559.14	0
129456	0	182.54	83.01	0	639.97	49.49
129460	0	351.67	292.24	251.97	1788.28	152.57
129466	0	458.41	0	141.96	1497.69	177.58
129472	0	185.55	146.74	115.45	1069.4	0

129494	0	420.25	286.04	160.08	2171.78	0
129502	0	122.11	59.06	145.23	1313.17	77.34
129510	0	203.6	116.05	143.41	1566.34	51.52
129518	0	148.52	72.34	225.88	2522.93	148.07
129530	0	109.6	0	112.18	1019.1	64.14
129532	0	82.56	35.62	21.15	693.52	52.31
129542	0	132.82	0	218.89	2168.34	75.73
129546	0	350.78	199.55	201.2	2241.89	232.44
129552	0	264.47	203.01	160.54	1887.68	103.63
129556	0	173.73	98.78	285.84	2804.8	160.32
129564	0	0	0	70.69	1600.72	62.48
129567	0	293.76	0	134.99	1879.71	112.42
129569	0	116.71	0	85.72	1468.99	58.84
129571	0	309	241.72	185.52	2362.27	228.86
129575	0	128.13	78.38	112.25	1200.69	83.21
129577	0	405.87	161.88	188.84	1676.34	113.93
129583	0	233.59	154	350.31	2424.74	101.99
129587	0	95.55	117.7	127.05	1535.81	84.92
129589	0	0	0	0	39.43	0
129605	0	30.84	106.21	44.43	1168.9	56.56
129607	0	264.85	183.36	117.69	1694.34	113.89
129609	0	69.83	0	0	185.17	0
129611	0	137.63	0	0	1049.42	32.3
129613	0	188.14	0	47.91	942.42	49.08
129615	0	62.34	0	0	1496.9	72.67
129621	0	39.41	69.18	0	850.45	27.76
129629	0	114.49	100.52	27.7	1044.9	0
129633	0	39.44	0	0	1432	146.01
129635	0	234.58	0	121.22	1762.47	0
129639	0	0	0	0	1211.62	0
129643	0	36.75	122.03	98.65	1353.94	104.02
129647	0	262.58	0	0	1127.14	0
129651	0	307.79	58.48	95.09	1294.34	76.77
129654	0	138.03	60.24	0	1581.35	0
129660	0	218.98	94.26	199.17	1540.41	0
129664	0	0	0	57.17	882.84	0
129670	0	99.41	0	96.43	1455.66	0
129676	0	328.01	362.69	247.6	2251.28	165.13
129687	0	361.22	383.64	296.29	2524.58	0
129699	0	62.65	56.01	0	615.83	32.33
129705	0	0	93.62	0	1225.24	94.02
129707	0	541.38	284.32	207.02	2624.41	359.87
129711	0	236.28	0	189.83	1996.64	61.06
129713	0	51.2	0	0	1565.83	0
129715	0	0	0	0	1733.63	0
129721	0	567.2	0	0	1277.79	0
129723	0	116.19	115.18	0	826.57	89.36
129727	0	64.3	120.05	105.49	1146.56	64.96
129729	0	312.82	114.39	92.74	1499.38	164.98
129739	0	261.56	0	0	1356.44	0

129743	0	0	78.93	0	962.81	0
129745	0	0	0	56.7	1104.11	85.24
129747	0	36.53	0	60.55	1011.57	0
129749	0	0	0	0	647.62	0
129753	0	0	0	39.95	1225.64	77.46
129776	0	131.85	43.53	0	740.93	22.08
129786	0	246.7	41.91	68.93	980.56	89.8
129792	0	0	0	107.79	1445.32	104.56
129794	0	62.47	0	0	800.97	0
129796	0	0	116.73	102.21	1629.09	0
129798	0	0	0	62.25	1060.49	0
129802	0	78.49	156.84	121.12	2334.44	109.63
129804	0	105.77	70.44	56.61	1374.57	0
129806	0	166.18	0	204.86	1529.35	50.49
129808	0	201.84	0	427.76	3046.36	281.29
129812	0	248.27	71.09	195.01	1689.6	123.2
129814	0	181.78	152.52	59.25	1431.89	88.08
129816	0	212.97	0	335.69	2993.52	296.04
129820	0	76.19	0	88.02	1373.74	74.43
129822	0	0	0	0	1287.39	0
129824	0	86.04	32.34	177.54	1271.25	86.7
129826	0	115.59	79.37	84.67	1145.31	39.05
129828	0	102.93	0	0	1022.63	0
129835	0	22.96	145.66	429.32	2978.14	189.95
129839	0	507.74	0	0	1889.98	0
129848	0	49.48	62.03	62.99	948.46	24.85
129860	0	249.56	0	0	1399.3	0
129862	0	211.93	18.29	83.69	915.62	21.91
129866	0	124.7	88.16	0	1724.47	0
129868	0	138.67	0	47.81	665.47	0
129870	0	26.25	0	135.92	1215.78	40.07
129881	0	0	73.06	47.74	1758.27	0
129889	0	347.41	218.4	0	1678.94	0
129891	0	167.05	109.92	106.84	1591.77	42.09
129897	0	216.59	245.61	137.27	1451.74	55.48
129901	0	170.14	163.66	72.8	1715.32	171.07
129909	0	184.87	0	247.91	2150.39	99.29
129913	0	498.08	241.43	409.18	2334.58	61.5
129915	0	164.23	0	53.4	1943.81	83.76
129916	0	393.67	0	255.24	2286.37	54.38
129922	0	34.34	60.34	30.4	272.68	5.82
129924	0	0	0	38.25	813.62	0
129926	0	0	64.53	201.08	1501.57	0
129933	0	158.06	181.79	62.38	1160.64	34.62
129937	0	275.22	410.79	280.49	2684.18	196.08
129939	0	284.28	0	0	2950.7	0
129947	0	132.38	0	40.93	1169.44	54.57
129953	0	128.91	128.27	91.75	974.22	32.04
129961	0	329.83	268.45	144.51	2931.26	0
129965	0	198.88	0	0	1210.52	0

129970	0	328.89	0	247.39	2105.76	45.34
129972	0	206.27	0	12.98	1176.05	34.61
129974	0	151.46	184.25	77.98	734.22	67.67
129978	0	49.56	32.45	0	712.01	81.3
129980	0	27.02	11.71	24.29	526.07	37.76
129984	0	30.31	0	0	1122.33	66.68
129986	0	0	0	0	1608.02	0
129988	0	275.06	73.09	0	1169.76	0
129990	0	0	0	56.34	935.63	0
129994	0	377.36	386.66	163.17	1818.48	240.52
129999	0	0	118.34	105.3	1690.38	0
130001	0	381.07	0	69.67	2131.59	0
130003	0	96.72	62.94	61.78	1121.39	36.31
130011	0	544.12	0	183.28	2922.57	132.97
130017	0	283.1	0	190.46	1998.16	0
130023	0	0	0	0	530.88	0
130025	0	169.1	217.72	0	1431.17	125.24
130029	0	91.04	0	0	1077.76	0
130031	0	0	0	0	702.21	0
130041	0	0	151.94	79.07	2356.89	254.57
130043	0	0	0	113.11	1296.08	0
130055	0	144.81	0	220.27	2406.63	0
130061	0	50.42	0	0	1488.31	0
130085	0	134.5	277.16	85.12	1885.21	0
130087	0	212.8	333.32	0	1812.41	88.6
130098	0	195.12	146.61	269.9	2405.86	80.99
130100	0	138.39	165.03	79.45	1094.09	0
130104	0	211.71	0	0	1110.93	41.04
130108	0	377.44	0	250.71	2776.66	101.04
130110	0	33.18	0	91.67	981.86	75.57
130114	0	473.7	53.6	266.57	2299.05	0
130116	0	208.33	0	114.11	1559.62	86.01
130124	0	96.58	270.75	77.5	1411.76	0
130127	0	418.35	226.4	144.3	1645.93	100.39
130130	0	44.82	176.34	51.4	809.46	0
130133	0	187.63	162.28	114.74	1448.96	30.71
130137	0	227.45	206.85	163.56	1541.51	130.72
130140	0	94.45	116.12	165.17	1629.29	0
130142	0	285.69	120.9	251.16	1705.41	161.04
130145	0	269.11	75.09	272.57	1664.65	99.39
130153	0	299.55	145.63	186.72	1703.91	107.36
130157	0	57.59	119.83	37.68	1380.16	35.79
130159	0	0	0	0	755.28	0
130163	0	123.16	0	0	633.19	0
130165	0	523.79	269.94	305.16	2891	163.09
130167	0	70.17	85.4	62.19	1164.71	37.77
130169	0	253.44	187	163.01	2048.67	175.18
130171	0	106.77	166.05	0	2772.26	309.03
130173	0	0	102.04	54.87	1580.94	47.08
130175	0	323.37	296.37	441.96	2839.87	341.2

130221	0	250.97	381.72	73.69	2271.84	0
130224	0	0	303.8	121.61	1729.74	0
130229	0	0	0	0	1258.42	114.25
130252	0	139.82	115.57	100.36	1540.09	133.38
130254	0	140.92	302.26	178.69	1554.04	59.01
130274	0	321.17	120.56	128.3	2506.06	123.81
130276	0	81.8	0	0	1034.22	0
130280	0	122.3	113.45	84.16	1451.84	0
130292	0	118.97	0	0	1627.64	0
130296	0	259.73	188.44	178.49	2048.89	183.11
130321	0	349.96	135.62	52.64	1370.86	0
130329	0	0	0	44.32	989.5	0
130337	0	18.89	0	0	495.59	0
130345	0	386.36	291.58	151.54	3130.48	201.34
130359	0	211.25	75.19	0	780.89	0
130361	0	41.74	0	0	531.5	0
130367	0	317.21	0	77.44	1649.73	0
130428	0	74.64	0	53.3	1263.57	0
130432	0	326.72	0	0	2156.31	0
130436	0	92.31	0	0	1705.53	0
130438	0	262.97	0	0	1051.9	0
130453	0	272.3	0	174.28	1709.41	148.79
130455	0	194.02	191.36	447.85	2930.67	139.72
130457	0	119.83	100.83	54.7	1186.44	73.99
130461	0	0	20.98	0	401.5	0
130465	0	216.6	192.93	189.41	1820.99	161.32
130467	0	196.53	180.27	231.61	3404.19	290.83
130473	0	297.07	0	0	1211.43	97.61
130476	0	90.47	0	0	1348.4	0
130478	0	246.28	0	0	1336.93	81.51
130482	0	48.63	0	0	1693.88	0
130488	0	711.69	143.2	300.18	2478.26	153.75
130490	0	187.38	0	0	1153.43	88.57
130503	0	0	114.84	0	2597.25	193.61
130505	0	142.77	208.65	226.23	3342.91	80.67
130509	0	42.75	0	0	3552.96	196.11
130511	0	79	0	0	480.59	0
130517	0	0	0	0	536.41	0
130520	0	73.13	130.5	203.79	2840.95	99.69
130524	0	0	47.47	14.92	295.95	0
130526	0	72.75	0	0	1137.23	102.53
130570	0	111.59	160.8	172.26	1253.77	187.71
130576	0	337.38	37.01	0	1924.77	0
130578	0	113.73	83.53	127.25	1577.16	101.82
130612	0	365.36	446.46	416.66	2620.97	417.82
130622	0	0	0	0	700.21	0
130626	0	145.61	345.98	172.37	2165.89	179.99
130634	0	516.57	227.63	0	538.35	0
130640	0	267.4	0	47.82	1335.97	50.49
130647	0	36.38	0	0	537.09	0

130649	0	0	65.13	0	610.14	0
130657	0	0	0	0	475.98	0
130666	0	0	0	0	1933.77	0
130696	0	0	0	0	1569.52	0
130712	0	183.22	54.88	0	839.13	0
130714	0	511.13	182.25	101.81	1741.68	0
130720	0	0	0	113.46	338.92	0
130734	0	0	0	132.07	2298.18	97.43
130752	0	126.64	361.3	152.3	1727.02	96.65
130754	0	352.83	0	61.06	1824.89	79.96
130782	0	463.65	452.35	501.14	3071.15	129.03
130784	0	222.44	422.17	489.05	3046.05	269.08
130794	0	0	0	28.19	604.57	0
130805	0	0	0	0	1022.92	0
130921	0	67.79	293.51	149.11	1191.02	98.77
130923	0	214.18	72.93	0	1565.44	0
130925	0	0	0	0	1340.69	96.83
130927	0	68.98	385.85	0	2737.39	0
130931	0	60.18	289.07	0	2876.8	0
130935	0	94.75	88.89	0	141.62	43.13
130937	0	0	0	0	871.57	0
130975	0	355.55	0	0	83.89	0
131019	0	319.29	237.07	83.8	1673.53	109.36
131021	0	157.62	15.48	133.16	1183.49	0
131033	0	0	71.22	0	578.65	0
131059	0	0	313.3	0	2254.11	0
131074	0	51.14	28.74	0	563.48	0
131076	0	114.8	0	0	1587.35	0
131086	0	0	0	0	589.1	0
131124	0	440.1	184.56	454.08	2616.35	114.71
131139	0	128.37	173.83	0	645.06	0
131155	0	0	0	49.32	1145.84	0
131199	0	241.92	49.6	0	1093.59	0
131213	0	0	0	0	863.02	0
131256	0	54.29	0	0	1929.95	0
131258	0	0	0	0	903.87	0
131346	0	0	0	0	35.38	0
131370	0	224.41	142.9	52.19	2086.21	246.58
131426	0	320.34	586.5	624.63	3568.94	0
131507	0	399.56	0	88.49	821.54	0
131662	0	151.99	268.75	315.8	2547.66	0
131764	0	0	35.89	37.79	400.18	0
131776	0	87.73	0	0	2111.12	0
131809	0	56.76	202.52	0	333.95	0
131847	0	44.29	426.23	123.93	2205.55	146.99
131897	0	67.93	120.72	110.69	1826.72	0
131915	0	87.75	0	392.11	2324.94	0
131964	0	0	0	0	783.45	0
133096	0	82.46	0	124.34	1882.04	139.82
133203	0	151.56	0	27.34	1746.85	0

133260	0	218.51	0	225.6	1804.54	135.14
133264	0	35.31	239.2	295.47	2193.24	126.21
133320	0	0	0	74.95	1126.24	0
133327	0	108.88	210.38	248.43	2101.1	129.64
133375	0	120.52	321.03	166.07	2309.86	140.35
133377	0	320.99	448.28	242.45	4067.65	163.46
133381	0	161.87	0	174.09	2131.56	117.2
133406	0	0	0	0	964.2	0
133582	0	89.5	0	91.89	1885.68	76.95
133607	0	115.94	99.94	0	965.7	184.74
133609	0	364.41	252.29	94.3	1105.76	0
133612	0	420.29	573.38	138.26	4000.9	356.94
133617	0	142.04	130.38	69.55	1032.16	0
133636	0	377.78	324.82	464	2956.11	215.5
133640	0	155.9	210.94	179.53	2308.73	0
133642	0	206.84	407.6	501.74	4320.29	216.3
133648	0	75.48	386.77	391.32	2811.64	159.5
133654	0	0	0	0	1118.68	69.7
133662	0	231.65	190.3	189.88	1797.39	146.39
133677	0	192.92	101.76	193.38	2623.1	408.53
133687	0	126.69	0	64.79	1281.82	41.26
133743	0	201.15	237.06	278.23	2763.62	282.55
133797	0	146.23	214.78	166.42	2183.43	185.66
133803	0	108.86	128.43	220.47	2195	363.49
133939	0	104.76	158.08	38.12	1736.95	67.32
133960	0	120.35	176.04	51.97	992.42	100.59
133988	0	0	0	0	268.48	0
134014	0	0	0	0	889.95	0
134061	0	178.22	122.83	152.62	1880.39	82.94
134113	0	56.56	67.35	21.58	984.04	0
134114	0	187.18	191.07	260.1	2136.74	169.75
134146	0	0	92.51	237.1	2134.43	63.45
134150	0	59.83	27.97	0	603.32	0
134165	0	55.26	0	67.24	986.29	0
134169	0	270.19	0	38.46	969.44	0
134179	0	55.27	59.97	18.1	502.94	12.95
134181	0	156.45	88.08	30.28	1050.22	73.59
134185	0	169.24	138.81	65.66	1524.93	0
134272	0	133.15	0	48.04	610.44	74.83
134274	0	307.81	179.33	0	1953.89	236.43
134282	0	0	0	0	927.45	0
134284	0	0	0	0	305.18	0
134286	0	234.46	233.73	111.45	1811.37	182.4
134287	0	249.8	259.19	126.63	1148.22	56.25
134305	0	120	113.36	110.32	1027.65	69.06
134322	0	163.34	132.49	131.71	1452.1	59.3
134330	0	83.72	0	43.6	800.38	0
134350	0	176	151.8	75.14	1510.21	34.06
134354	0	405.28	333.45	386.25	3014.23	254.21
134358	0	116.37	83.06	167.91	1683.1	61.26

134385	0	165.37	77.11	73.01	1522.4	0
134395	0	374.46	322.25	179.28	1827.48	117.02
134492	0	245.11	176.24	217.43	1570.03	0
134545	0	104.83	0	84.63	1126.15	0
134561	0	99.01	0	325.51	2176.87	104.09
134663	0	180.5	349.76	292.42	2370.02	165.73
135584	0	121.57	0	124.15	1517.43	0
135586	0	333.95	244.94	243.49	2169.29	0
135592	0	646.36	255.14	261.08	2095.89	0
135593	0	390.04	0	70.03	1292.53	0
135595	0	328.93	365.63	574.74	4615.6	534.22
135613	0	406.8	0	166.11	3459.45	258.05
135623	0	432.49	0	74.21	1553.59	0
135627	0	313.96	0	107.03	1193.34	0
138981	0	54.43	0	50.76	1241.62	134.35
139163	0	68.93	0	73.94	701.09	16.1
139672	0	143.17	160.3	37.52	1566.76	45.94
139706	0	203.83	157.27	156.52	1109.21	73.02
139723	0	0	37.4	0	1362.46	44.72
139739	0	0	0	16.32	1043.48	0
139751	0	154.29	127.58	164.94	1742.07	0
140166	0	58.71	0	124.09	1920.79	71.72
140333	0	227.49	312.49	161.08	2086.12	161.33
141617	0	133.5	110.82	41.49	919.15	39.47
141626	0	503.38	436.23	547.78	2232.1	157.01
141641	0	260.94	210.46	94.92	1510.06	141.21
141643	0	299.45	198.98	393.03	2110.45	228.39
141644	0	141.87	173.14	147.58	1585.93	0
141645	0	354.49	296.29	94.53	1581.95	120.89
141646	0	114.51	79.59	0	904.58	74.63
141696	0	232.06	0	0	935.72	53.32
141873	0	121.45	94.96	68.35	1159.47	77.34
141970	0	110.66	185.57	162.51	1824.48	172.72
142408	0	0	0	0	107.39	0
142670	0	67.41	0	40.18	646.19	13.12
142681	0	166.68	79.52	60.99	824.08	53.55
143242	0	77.37	49.12	32.42	428.86	14.69
143516	0	104.33	83.03	120.93	1184.75	32.18
143535	0	243.92	223.25	131.57	1449.05	162.96
143537	0	67.9	56.59	24.85	671.76	0
143542	0	94.44	98.8	159.83	2331.02	0
143543	0	107.48	0	0	995.8	0
143549	0	102.07	0	181.5	2223.85	154.25
143710	0	9.44	29.14	17.96	277.61	20.12
144332	0	174.85	246.11	222.79	1766.6	232.39
144367	0	156.22	111.89	0	1125.26	94.42
144510	0	0	103.16	51.78	1039.37	43.53
144512	0	0	0	29.92	469.51	0
144517	0	37.39	51.03	0	1651.77	102.88
144525	0	905.84	0	0	682.98	0

144606	0	30.53	26.65	0	598.06	8.52
144676	0	0	0	0	735.14	0
144677	0	33	135.86	45.99	1618.78	77.78
144687	0	124.36	73.82	0	765.4	55.59
144882	0	72.41	0	111.56	1508.53	59.06
144886	0	187.27	132.18	106.45	1532.92	142.52
144888	0	128.5	40.92	88.4	926.99	65
144926	0	50.52	81.5	49.91	659.9	18.31
144974	0	49.36	93.74	0	817.03	0
145014	0	435.47	334.46	333.49	3174.88	188.69
145046	0	67.82	0	181.61	2708.91	59.48
145127	0	190.55	162.19	0	1980.71	48.74
145196	0	107.63	93.14	174.17	2136.81	128.48
145255	0	222.2	0	0	2048.2	0
145284	0	105.96	145.42	161.8	1703.47	40.08
145505	0	50.91	0	0	701.14	0
145640	0	0	38.37	0	961.89	0
145739	0	67.2	0	0	691.99	28.5
146082	0	247.6	130.92	135.5	1886.44	84.99
146217	0	21.83	0	0	187.72	0
146343	0	182.59	170.87	48.51	1348.01	118.86
146498	0	122.83	81.16	34.55	731.81	42.14
146512	0	0	20.87	0	460.79	21.01
146718	0	128.88	185.05	46.45	534.07	41.88
146721	0	69.15	95.07	63.47	1008.43	16.96
146800	0	272.58	158.41	150.9	1406.3	155.9
146801	0	132.6	190.72	86.55	1011.2	92.13
146809	0	280.8	225.9	93.7	1872.59	203.05
147140	0	229.1	76.75	62.04	1118.58	49.37
147224	0	20.17	0	35.3	490.64	27.32
147234	0	130.15	65.52	143.82	1959.24	159.21
147368	0	179.54	102.14	15.34	918.55	0
147486	0	25.61	0	76.1	878.83	49.32
147554	0	17.74	24.2	41.87	426.17	33.89
147555	0	154.29	124.4	225.34	1194.72	0
147567	0	50.08	83.98	101.6	694.84	0
147584	0	269.97	373.31	235.22	2028.19	0
147697	0	63.72	53.67	0	635.48	25.38
147700	0	77.8	0	69.53	1389.31	61.38
147705	0	45.5	29.15	66.1	1417.48	114.04
147811	0	255.21	368.92	215.15	2461.37	81.3
151601	0	98.98	113.63	0	2610.68	196.23
151757	0	172.7	376.21	272.82	3426.67	110.49
152605	0	80.13	0	0	1122.79	0
153238	0	132.75	0	243.32	2042.83	108.92
154341	0	302.96	256	273.6	2397.72	160.67
154382	0	115.9	470.24	77.92	2161.96	0
155627	0	101.65	0	43.3	1038.45	0
155635	0	81.55	48.3	23.93	637.12	16.95
155636	0	182.03	0	113.29	1022.91	0

155927	0	0	0	0	398.66	0
155955	0	259.76	328.65	211.96	1882.7	130.59
155960	0	145.28	89.48	48.86	551.27	0
156001	0	124.06	81.21	66.33	836.4	67.67
156027	0	110.03	53.11	72.69	590.82	0
156147	0	130.23	0	75.14	1348.3	40.98
156212	0	283.29	120.47	127.51	1558.27	159.66
156252	0	218.29	208.14	101.88	1470.72	141.8
156360	0	343.92	0	0	1455.11	415.25
156366	0	173.59	0	0	1223.58	0
156452	0	197.17	155.21	89.16	1286.78	43.4
156552	0	18.73	36.27	0	331.54	0
156720	0	188.4	84.17	61.12	889.52	55.08
157181	0	48.25	105.86	78.44	1252.65	37.12
157290	0	14.4	17.71	25.62	225.07	6.16
157329	0	132.83	125.15	147.28	1808.3	37.86
157342	0	320.03	392.18	305.51	3180.09	260.21
157344	0	133.16	136.7	137.2	1371.63	113.56
157348	0	88.8	145.82	61.36	631.97	41.23
157404	0	196.17	102.15	106.77	1096.55	26.63
157472	0	32.32	40.85	0	815.59	0
157496	0	252.64	120.33	264.93	1813.52	63.8
157517	0	0	162.32	0	292.9	0
157582	0	156.73	0	276.68	2424.6	84.55
157646	0	139.97	134.53	80.98	1847.33	96.25
157691	0	18.85	45.28	0	683.83	0
157696	0	46.68	0	0	331.98	0
157711	0	126.68	106	46.2	907.94	0
157827	0	63.58	0	237.39	2110.86	136.12
157970	0	100.77	37.95	50.01	481.64	26.52
158133	0	37.64	0	12.99	617.34	37.47
158904	0	116.27	0	57.42	1055.75	52.41
158905	0	83.32	90.65	64.83	816.46	66.2
158907	0	160.69	86.6	84.77	995.01	0
159008	0	196.49	65.22	23.2	627.09	0
159321	0	177.12	208.44	237.46	3176.14	244.17
159348	0	108.23	42.5	141.27	1443	79.8
159352	0	122.71	148.92	0	1453.71	120.32
159355	0	308.45	335.44	287.88	2462.87	214.01
159357	0	205.32	195.48	237.52	2326.82	146.61
159363	0	379.11	281.88	276.6	2523.82	163.05
159373	0	85.06	130.19	99.36	1527.29	134.53
159612	0	0	0	0	927.11	0
159623	0	445.54	221.66	303.45	2788.41	165.32
159656	0	19.96	59.97	9.35	784.44	0
159761	0	17.63	0	0	963.4	0
159762	0	113.5	0	34.57	1131.08	0
159939	0	201.48	139.99	58.23	1570.28	13.67
159973	0	334.05	130.17	90.18	1173.8	30.43
160112	0	51.09	187.28	85.67	1568.33	0

160113	0	7.37	0	49.92	846.38	0
160161	0	300.97	265.24	311.6	2415.35	160.15
160223	0	185.08	224.11	121.16	1389	131.37
160254	0	135.93	100.73	80.22	885.96	63.32
160472	0	86.14	0	0	1281.01	0
160489	0	300.94	249.9	264.34	2461	350.3
161155	0	0	0	0	1034.05	0
161156	0	0	0	0	1011.37	0
161876	0	203.29	146.29	104.64	1505.98	93.77
161897	0	50.48	0	103.38	1472.61	54.22
161920	0	106.4	119.4	79.17	1054.46	79.55
162906	0	77.96	43.49	42.51	956.48	76.53
162971	0	79.1	124.79	108.73	1450.43	118.12
163306	0	189.89	0	64.95	722.64	0
163571	0	0	19.34	11.25	438.71	29.33
163608	0	0	51.96	35.69	1006.63	0
163617	0	0	0	0	934.51	0
163641	0	33.35	73.76	27.08	965.09	32.75
163688	0	40.58	0	63.91	1242.78	46.78
163848	0	90.8	115.98	49.01	1355.37	0
163910	0	546.41	0	0	2289.13	0
164715	0	144.09	126.35	64.78	1200.31	82.77
164717	0	192.74	0	224.72	2618.27	26.97
164724	0	317.88	214.73	74.9	2318.15	276.63
164726	0	346.41	392.89	313.39	2486.59	78.64
164730	0	317.48	230.04	187.03	2629.68	174.09
164873	0	83.67	0	0	1308.5	86.06
165102	0	0	34.04	0	692.89	29.65
165103	0	318.23	324.55	433.25	3381.19	177.65
165132	0	32.77	0	52.23	621.23	0
165287	0	186.23	179.44	105.53	1955.04	124.41
165930	0	642.53	320.18	283.72	2150.87	51.41
167014	0	18.67	0	0	108.98	0
168247	0	6.62	49.46	11	478.63	0
169657	0	113.18	100.48	21.24	613.51	45.3
169722	0	197.48	275.49	173.08	1993.94	178.35
169938	0	39.25	26.46	0	450.18	0
169940	0	87.85	52.44	23.99	457.96	0
169950	0	170.31	159.64	128.61	1221.35	95.35
171263	0	0	0	0	372.18	0
171301	0	0	0	0	553.12	0
171330	0	89.25	90.4	49.51	1012.74	44.15
171543	0	0	104.6	29.13	1109.53	0
171611	0	333.87	0	0	1288.86	0
171653	0	279.54	134.07	0	1284.76	100.14
171679	0	20.49	66.98	52.57	1159.22	13.05
171714	0	31.37	121.19	59.99	1123.68	31.86
171744	0	29.95	0	0	792.07	0
171768	0	85.53	220.98	54.62	1208.28	55.95
171777	0	104.76	289.42	222.63	2273.2	268.21

171781	0	109.76	0	31.13	1480.45	0
171794	0	0	0	0	203.35	0
171822	0	0	141.27	0	899.83	0
171915	0	0	0	0	1019.25	0
194263	0	172.51	178.55	186.45	1275.24	85.9
194332	0	413.9	456.11	545.87	2997.9	331.63
194407	0	0	0	0	382.38	0
194621	0	302.7	176.72	114.75	1331.33	59.32
194701	0	0	0	0	1769.93	0
195034	0	320.51	385.63	302.31	2041.65	0
195045	0	363.52	421.87	235.19	1792.36	161.32
195051	0	93.75	278.53	300.02	1903.37	184.88
195053	0	391.18	630.85	437.53	3283.62	279.63
195219	0	0	0	0	582.57	0
195228	0	358.76	0	0	1858.09	0
195230	0	0	297.06	302.95	2296.12	0
195780	0	281.6	207.94	96.46	1101.06	106.15
195853	0	120.49	122.54	0	913.41	63.7
195893	0	393.63	322.14	182.35	1778.12	125.4
195906	0	490.37	293.04	0	1496.7	93.89
196022	0	273.02	239.7	200.85	1951.91	174.07
196032	0	0	0	0	363.74	0
196072	0	271.43	154.5	117.54	1047.28	75.38
196073	0	304.44	249	309.84	3154.42	226.49
196115	0	179.38	171.56	155.98	1359.71	113.31
196119	0	315.93	325.73	287.98	2086.3	236.63
196122	0	199.47	243.82	235.76	2011.17	170.46
196137	0	224.05	286.52	210.63	2214.15	203.77
196152	0	184.16	103.47	94.25	1087.12	96.26
196160	0	378.19	303.59	181	1823.98	204.05
196167	0	245.39	182.89	158.45	2083.77	0
196170	0	275.81	140.2	67.32	1892.31	55.13
196174	0	274.67	186.23	108.09	1228.04	124.36
196177	0	352.85	272.84	196.6	1514.21	113.87
196188	0	336.61	237.51	131.16	1494.64	174.96
196189	0	272.31	162.02	226.97	2387.7	208.24
196193	0	0	0	20.61	503.08	22.75
196199	0	289.11	190.67	78.69	842.91	82.81
196201	0	277.87	251.82	211.68	1770.87	197.32
196502	0	34.08	187.94	0	1028.69	0
196809	0	0	0	41.34	831.53	0
196810	0	0	0	26.54	1445.83	91.23
196830	0	176.59	148.54	67.49	1750.53	139.48
197881	0	80.4	0	0	1001.01	0
197916	0	91.86	99.5	141.94	1972.54	43.32
197919	0	94.55	91.22	85.69	1642.9	49.74
198001	0	70.33	0	0	1441.52	52.97
198005	0	129.62	0	31.44	744.62	0
198022	0	162.19	148.12	82.24	1900.49	60.69
198024	0	0	75.39	0	1145.22	0

198025	0	286.86	206.15	137.74	1631.31	132.99
198026	0	327.22	414.18	319.83	3594.77	304.11
213771	0	61.68	0	0	1552.81	0
213846	0	386.17	324.03	309	2172.93	93.45
213909	0	555.65	466.44	633.29	3951.44	384.23
213911	0	234.12	208.51	0	1666.82	80.21
213915	0	388.03	461.11	610.34	4393.45	0
213926	0	221.32	139.09	0	1550.54	0
213928	0	214.33	187.88	0	1105.36	40.47
213932	0	120.82	61.72	29.4	745.74	0
213937	0	316.51	170.76	200.69	1537.71	132.97
213944	0	200.46	275.25	155.37	1407.31	0
213995	0	0	0	40.3	715.83	25.66
214008	0	380.56	276.13	236.62	2916.1	273.32
214012	0	419.07	0	0	2748.41	0
214027	0	266.04	159.73	0	1145.12	83.39
119097	1	242	325.41	0	1379.81	120.69
119101	1	411.37	0	0	1173.33	0
119112	1	106.41	108.61	101.78	1413.76	0
119117	1	0	0	0	0	0
119120	1	0	0	0	363.46	0
119122	1	0	0	0	0	0
119127	1	0	105.84	0	1623.77	152.88
119132	1	261.94	0	0	980.05	0
119133	1	0	0	0	2175.03	171.39
119145	1	31.05	298.98	207.86	3057.73	299.4
119149	1	204.01	146.48	123.99	1278.12	111.39
119153	1	0	0	0	335.82	0
119154	1	171.86	86.79	35.88	555.55	0
119165	1	119.6	363.92	0	1553.69	0
119167	1	0	0	0	86.81	0
119185	1	770.03	377.85	230.91	2315.54	100.55
119186	1	112.88	0	94.41	777.18	0
119187	1	0	0	0	807.89	0
119195	1	64.57	0	0	664.72	0
119198	1	0	26.4	0	439.49	0
119212	1	0	247.04	251.72	3115.68	228.11
119214	1	0	0	0	763.43	0
119217	1	0	147.58	93.22	2628.72	119.42
119218	1	0	170.02	195.3	2093.31	144.32
119222	1	0	0	0	1028.9	0
119224	1	40.94	0	25.6	265.98	0
119236	1	0	0	0	796.44	0
119244	1	558.31	0	0	401.59	0
119258	1	0	51.66	1525.41	612.26	230.82
119259	1	0	0	0	0	0
119262	1	179.88	0	0	409.64	0
119264	1	55.6	0	78.38	826	30.84
119266	1	177.12	201.67	203.31	1576.88	150
119278	1	0	0	257.4	1648.16	0

119279	1	0	0	0	177.46	0
119283	1	0	0	0	1009.36	0
119284	1	0	0	0	475.06	0
119287	1	0	0	0	1364.27	0
119300	1	122.59	0	112.66	2120.25	0
119306	1	0	883.66	4988.91	2062.07	728.21
119377	1	215.3	128.57	24.15	768.46	0
119383	1	117.45	72.39	93.01	317.41	0
119389	1	0	46.18	27.17	318.48	0
119408	1	0	15.48	13.49	154.5	0
119418	1	0	23.02	0	121.13	0
119421	1	0	95.71	113.4	1754.27	113.87
119449	1	0	0	106	2960.49	0
119540	1	0	0	0	52.64	0
119562	1	0	0	0	197.82	0
119574	1	26.65	31.67	0	624.02	0
119745	1	302.43	171.34	116.96	723.98	37.52
119792	1	0	0	0	829.59	0
119804	1	0	0	0	201.26	0
119815	1	0	0	0	629.11	0
119845	1	150.36	122.66	156.3	1305.41	139.03
120018	1	0	0	0	432.56	0
120247	1	0	0	0	44.43	0
120274	1	0	31.33	39.27	317.34	0
120908	1	0	30.3	0	310.67	0
120926	1	0	0	0	104.59	0
120952	1	0	0	105.09	1081.19	0
120954	1	0	0	0	13.95	0
121015	1	0	0	0	0	0
121069	1	0	0	0	909.19	0
122191	1	0	0	0	1083.31	0
122222	1	74.65	109.64	100.79	1296.66	71.51
122226	1	0	0	0	101.65	0
122228	1	0	0	0	703.51	0
122230	1	0	0	0	213.17	0
122257	1	0	0	0	189.26	0
122263	1	0	0	0	1328.61	0
122267	1	45.94	0	0	619.43	0
122274	1	396.21	0	0	177.15	0
122283	1	0	0	0	114.01	0
122414	1	0	0	0	474.46	0
122639	1	110.24	0	78.5	0	0
122640	1	0	0	0	713.93	0
122643	1	69.93	0	0	1448.27	0
122659	1	0	0	0	197.2	0
122679	1	0	0	0	0	0
122683	1	0	0	3151.33	0	0
122685	1	710.8	0	0	0	0
122738	1	0	0	0	416.46	0
122746	1	0	0	0	0	0

122768	1	0	0	0	326.56	0
122774	1	0	0	0	219.35	0
122792	1	0	0	0	290.43	0
122794	1	0	0	0	338.76	0
122796	1	0	0	0	377.98	0
122804	1	0	0	0	184.34	0
122810	1	767.39	0	0	2451.2	908.88
122812	1	0	0	0	258.81	0
122818	1	32.57	0	145.07	1718.24	0
122826	1	59.03	0	0	286.32	0
122857	1	92.31	0	0	648.36	0
122978	1	200.57	0	0	960.77	0
123018	1	0	0	0	412.8	0
123020	1	728.01	0	185.49	1022.96	0
123052	1	114.57	0	0	537.25	0
123064	1	0	0	0	0	0
123117	1	0	0	22.04	470.89	0
123594	1	241.39	0	0	1010.63	0
123608	1	129.76	0	0	784.57	0
123611	1	0	207.03	6268.86	1203.82	0
123619	1	118.84	0	0	421.98	0
123681	1	222.1	136.89	50.9	873.69	0
123765	1	0	0	0	0	0
123777	1	71.19	0	0	157.38	0
123834	1	476.18	0	1543.76	629.77	457.18
123855	1	0	0	982.12	0	0
123861	1	455.08	0	0	1046.12	0
123897	1	76.35	0	148.46	378.36	0
123947	1	86.49	0	0	202.64	0
124152	1	0	0	0	896.05	0
124178	1	0	0	0	149.72	0
124216	1	45.85	49.65	93.2	496.43	0
124345	1	129.73	97.55	0	351.02	0
124361	1	41.6	0	0	479.93	0
124414	1	0	0	0	251.77	0
124444	1	82.27	0	40.05	700.02	0
124446	1	58.55	0	0	305.98	0
124448	1	0	0	0	247.81	0
124511	1	202.66	0	0	501.34	0
124513	1	117.68	0	0	1244.11	0
124712	1	56.82	0	0	217.29	0
124716	1	0	0	0	418.42	0
124742	1	0	0	0	0	0
124757	1	0	0	0	113.94	0
124759	1	0	0	0	110.96	0
124760	1	0	0	0	140.1	0
124763	1	0	0	0	121.72	0
124767	1	0	0	0	109.35	0
124769	1	174.05	0	0	285.15	0
124771	1	0	0	0	147.6	0

124777	1	0	0	0	540.59	0
124779	1	0	0	0	166.22	0
124798	1	0	0	0	57.72	0
124800	1	134.35	0	0	874.69	0
125071	1	192.75	92.72	64.23	539.57	0
125128	1	0	0	0	173.16	0
125560	1	0	48.04	0	390.64	0
125842	1	17.69	0	40.26	570.02	0
125851	1	70.06	0	20.4	644.33	0
125859	1	0	0	0	0	0
125873	1	0	0	935.95	0	0
125875	1	0	0	0	356.03	0
125877	1	0	0	0	251.84	0
125887	1	0	0	0	23.5	0
125891	1	58.6	0	0	0	0
125895	1	0	0	0	0	0
125897	1	0	0	0	0	0
125899	1	0	0	0	0	0
125901	1	397.6	0	535.05	705.52	0
125903	1	0	0	0	80.64	0
125909	1	170.66	0	0	462.87	0
125911	1	373.55	0	0	454.7	0
125913	1	2377.11	494.15	0	0	0
125917	1	0	0	0	98.98	0
125921	1	40.81	11.19	0	625.67	0
125923	1	0	0	0	0	0
125929	1	637.99	60.06	0	0	0
125971	1	159.79	0	0	173.24	0
125979	1	0	0	0	33.02	0
125987	1	89.38	118.94	0	617.63	34.63
125988	1	0	0	0	379.01	0
125994	1	0	0	0	360.62	0
125998	1	0	0	0	543.98	0
126002	1	0	0	0	159.52	0
126024	1	0	60.55	0	469.98	0
126042	1	0	0	0	625.76	0
126067	1	107.89	0	0	1054.46	76.82
126073	1	179.86	0	0	359.13	0
126087	1	54.51	25.06	0	365.47	0
126099	1	47.47	0	0	215.68	0
126119	1	38.32	0	27.5	599.35	0
126121	1	0	0	0	180.09	0
126131	1	323.02	0	0	0	0
126153	1	0	0	0	253.84	0
126169	1	0	0	0	0	0
126184	1	88.14	0	0	0	0
126186	1	0	0	0	0	0
126207	1	0	0	0	356.89	0
126219	1	154.42	0	0	683.36	0
126784	1	0	0	0	662.39	0

126815	1	0	0	0	289.53	0
126817	1	44.48	0	0	672.79	0
126823	1	0	0	0	524.95	0
126829	1	76.51	0	35.68	974.56	0
126835	1	0	0	0	271.23	0
126849	1	0	11.67	0	90.97	0
126853	1	0	47.62	0	313.73	0
126857	1	0	0	73.18	532.66	0
126861	1	0	0	0	346.14	0
126865	1	0	138.37	0	1654.04	0
126871	1	21.36	0	49.75	676.65	57.48
126873	1	188.73	108.53	0	646.94	0
126875	1	0	0	42.36	458.29	0
126879	1	0	0	0	579.83	0
126881	1	103.7	41.62	69.13	652.85	0
126901	1	258.68	0	63.67	1139.79	0
126907	1	0	0	0	588.46	0
126911	1	0	0	0	158.24	0
126913	1	0	0	0	308.39	0
126917	1	0	0	0	188.66	0
126921	1	93.02	0	44.72	851.36	31.94
126925	1	53.38	0	14.59	489.34	19.11
126929	1	40.11	0	0	185.62	0
126939	1	0	0	0	1121.33	91.41
126942	1	0	39.76	0	357.28	52.45
126945	1	0	0	0	543.51	0
126947	1	0	0	0	0	0
126951	1	0	0	0	1787.94	260.79
126957	1	0	97.31	18.31	663.86	22.18
126959	1	524.28	0	25.62	1186.83	0
126961	1	48.35	0	27.76	698.55	0
126963	1	0	0	57.07	1052.57	0
126965	1	0	31.75	0	232.57	0
126967	1	306.57	104.89	0	1389.94	94.24
126973	1	0	0	0	0	0
126977	1	57.17	0	0	460.92	0
126981	1	0	0	0	0	0
126983	1	70.43	0	0	435.4	0
126985	1	0	0	0	624.32	0
126989	1	0	72.73	0	1243.04	0
126995	1	0	0	0	0	0
126999	1	0	0	0	204.12	0
127007	1	0	0	0	857.07	0
127009	1	0	0	0	1278.6	0
127011	1	0	0	0	1062.32	0
127015	1	0	0	426.41	3736.53	238.6
127017	1	0	0	0	619.06	0
127023	1	0	0	38.54	1905.85	0
127031	1	0	0	39.96	606.7	0
127035	1	52.36	0	0	1151.93	150

127043	1	0	0	0	721.27	0
127049	1	0	0	0	734.11	0
127051	1	594.07	0	0	1257.25	0
127067	1	318.9	69.2	0	1277.41	110.81
127069	1	299.74	0	128.1	1132.11	77.43
127551	1	212.18	0	0	2350.79	0
128064	1	0	169.7	122.77	1243.86	102.59
128452	1	148.62	0	0	364.65	0
128515	1	0	0	0	391.32	0
128614	1	0	0	0	461.92	0
128699	1	0	434.65	442.91	2812.48	60.55
128771	1	0	0	278.6	1648.25	48.79
128844	1	0	117.77	0	784.78	0
129214	1	0	0	0	0	0
129216	1	0	0	0	75.21	0
129224	1	0	944.24	0	1428.85	2252.2
129226	1	42.66	61.62	38.88	349.63	0
129234	1	17346.2	4932.63	0	625.92	0
129373	1	572.5	380.89	352.91	2922.32	0
129512	1	0	0	0	380.83	0
129619	1	172.55	0	0	778.23	0
130009	1	34.67	0	0	1356.11	0
130466	1	142.62	84.49	145.95	1550.93	86.92
130574	1	236.48	105.13	85.6	1623.25	168.13
130600	1	253.29	0	0	584.31	0
130979	1	82.1	0	0	475.19	0
134720	1	0	0	0	162.52	0
136668	1	87.52	117.1	18.86	677.65	0
136672	1	54.71	0	0	272.27	0
137640	1	18.47	0	0	349.78	14.21
139676	1	107.05	111.15	88.84	1382.51	142.7
139678	1	91.32	139.82	110.36	1292.84	101.09
141302	1	0	0	0	169.04	0
141624	1	30.25	62.2	0	436.9	0
141681	1	101.14	56.12	11.72	827.02	0
141972	1	54.03	89.64	0	844.22	22.97
142071	1	47.66	0	0	1013.07	0
143055	1	27.71	42.56	28.11	847.39	0
143518	1	45.57	51.74	32.54	365.6	0
144524	1	90.74	45.47	0	872.02	0
144531	1	0	115.41	73.77	1242.02	74.84
144574	1	91.34	103.09	36.33	1041.93	28.91
144740	1	0	43.43	36.32	567.61	0
145003	1	193.59	198.27	221.72	1893.94	144.49
145126	1	28.6	0	34.19	572.2	0
145195	1	85.82	57.69	77.37	1121.58	0
145282	1	210.01	0	106.02	3170.99	434.38
145557	1	188.53	0	0	636.52	0
145559	1	170.01	0	0	1045.55	0
145566	1	431.14	0	0	0	0

145571	1	0	0	0	0	0
145574	1	0	0	0	164.69	0
145576	1	0	0	0	331.1	0
145578	1	45.27	0	0	104.62	0
145593	1	725.97	785.75	3682.87	1742.21	429.66
145595	1	29.26	33.28	0	226.32	0
145610	1	412.51	0	1413.51	536.8	0
145611	1	477.32	0	1177.26	1610.76	0
145646	1	79.28	0	0	1063.78	0
145648	1	0	0	0	348.54	0
145660	1	28.79	0	0	444.6	0
145736	1	0	242.84	0	294.65	0
145781	1	198.61	0	0	0	0
145782	1	0	0	0	0	0
145794	1	121.25	0	52.06	423.05	0
145799	1	0	0	0	703.16	0
145933	1	0	97.37	0	595.47	0
145934	1	28.56	0	0	426.7	0
145936	1	0	0	0	198.39	0
146031	1	0	44.36	21.35	670.63	40.68
146320	1	19.01	78.08	0	662.51	39.15
146328	1	123.09	74.33	38.84	614.72	53.88
146507	1	0	0	0	566.04	0
146661	1	169.33	139.49	62.71	838.74	23.76
146770	1	147.88	0	0	660.26	39.52
146785	1	127.1	143.21	81.06	1236.47	26.4
146797	1	109.2	0	67.43	1238.04	48.79
147160	1	100.91	82.09	52.84	743.7	0
147238	1	0	0	0	943.87	0
147482	1	0	167.29	38.04	1069.64	0
147491	1	0	0	0	569.82	0
147638	1	175.02	102.34	105.83	694	27.9
147641	1	0	44.02	14.56	489.1	74.56
147642	1	98.31	151.85	0	1270.13	0
147687	1	61.33	149.73	81.41	957.58	103.46
147780	1	0	0	0	1009.72	10.65
147809	1	262.44	223.42	277.87	1977.82	43.72
151471	1	130.66	47.48	0	783.32	0
151851	1	157.9	175.66	147.13	1582.37	108.52
153434	1	0	0	0	325.53	0
153689	1	102.7	78.95	69.68	660.82	26.13
153696	1	80.64	33.59	50.92	634.27	0
153711	1	0	27.83	0	102.57	0
155622	1	70.27	0	22.8	788.51	37.13
155632	1	82.43	0	0	978.88	0
156155	1	0	0	28.33	582.87	0
156210	1	174.06	183.95	108.15	995.36	0
156350	1	41.34	77.66	176.17	2179.27	230.08
156390	1	135.52	132.59	85.74	1381.38	57.21
156423	1	263.38	136.05	121.07	1561.82	93.06

156557	1	0	0	0	522.47	0
156558	1	262.86	245.56	146.86	1212.53	0
156566	1	151.34	91.4	0	1097.67	22.01
156632	1	0	159.2	70.23	1258.7	0
157226	1	0	29.57	0	590.43	0
157347	1	65.17	42.47	21.85	212.2	0
157354	1	77.58	0	0	310.89	0
157405	1	152.12	87.69	86.55	1529.95	58.21
157432	1	33.16	42.27	0	361.96	0
157453	1	163.59	176.3	114.85	1561.22	130.72
157474	1	181.76	0	0	725.67	0
157493	1	0	0	0	272.55	0
157506	1	0	0	0	712.09	0
157508	1	289.43	0	0	0	0
157512	1	0	0	0	127.07	0
157531	1	0	0	0	168.38	0
157555	1	0	0	0	671.41	0
157559	1	110.61	0	0	148.56	0
157562	1	0	0	0	372.05	0
157647	1	155.51	170.95	248.79	2599.65	213.4
157665	1	117.91	215.36	90.57	978.93	86.71
157824	1	0	0	0	162.67	0
157987	1	54.83	53.67	0	300.9	0
158144	1	128.3	93.03	60.92	691.55	61.19
158801	1	34.13	9.56	0	1040.39	0
158806	1	197.81	149.52	68.72	1550.1	22.11
158995	1	80.27	74.69	40.27	822.65	39.78
159005	1	57.44	0	17.9	601.15	0
159007	1	0	0	0	275.14	0
159314	1	203.14	266.15	185.24	1379.23	0
159316	1	74.23	136.94	57.65	1102.72	48.44
159382	1	134.16	134.92	128.95	1657.11	106.21
159467	1	42.68	0	20.21	694.98	0
159624	1	0	26.2	0	444.58	0
159639	1	193.11	0	0	268.81	0
160391	1	50.17	0	0	531.57	0
160393	1	124.48	95.98	33.52	999.81	68.91
161063	1	352.75	189.4	293.51	2037.58	0
161064	1	131.12	217.43	0	1032.45	0
161068	1	0	0	0	452.37	0
161070	1	0	0	17.19	538.14	50.45
161071	1	0	0	0	393.95	54.93
161073	1	0	0	0	381.56	0
161871	1	28.3	67.44	46.68	1228.78	88.11
162291	1	99.77	131.77	0	923.62	29.62
162928	1	205.52	139.32	83.68	1386.56	79.58
164244	1	0	0	0	1152.69	0
164255	1	0	0	0	82.11	0
164258	1	156.32	76.84	21.36	298.85	0
164719	1	158.83	62.84	53.06	1245.62	29.61

164723	1	107.55	0	56.23	753.58	36.05
164727	1	179.58	134.66	0	1537.08	0
164855	1	137.61	33.77	39.36	491.11	41.21
165049	1	333.19	197.46	75.4	1542.14	100.73
165057	1	0	56.52	14.4	826.78	0
165084	1	115.91	93.99	0	1083.1	0
165097	1	0	451.21	197.1	3828.43	271.35
165192	1	148.15	163.16	116.47	1961.64	19.59
165521	1	16.6	39.29	0	385.48	0
166578	1	0	0	0	462.02	0
167093	1	206.04	167.21	157.42	1492.74	115.46
167153	1	170.31	0	0	0	0
167368	1	409.55	0	0	463.8	0
167760	1	0	0	77.22	1182.59	16.67
168246	1	24.3	0	12.3	321.21	0
168249	1	19.93	0	0	779.8	0
168612	1	0	0	0	433.87	0
168682	1	0	0	0	233.91	0
168841	1	70.29	103.38	95.3	979.13	44.31
168846	1	48.21	0	21.12	793.1	19.99
169897	1	205.05	296.16	213.52	2321.47	301.35
169904	1	282.48	351.49	71.44	1761.42	220.88
171225	1	255.34	140.95	67.65	953.11	0
171241	1	0	0	0	0	0
171242	1	0	0	0	528.82	0
171250	1	0	0	0	62.66	0
171252	1	0	0	0	0	0
171253	1	76.34	283.83	108.34	1363.37	71
171255	1	0	0	0	501.9	0
171256	1	0	0	0	0	0
171267	1	21.29	0	0	326.19	0
171268	1	0	0	0	0	0
171269	1	0	0	38.52	552.51	0
171270	1	64.95	0	0	237.32	0
171279	1	284.07	14.8	0	1199.9	0
171284	1	56.23	0	0	641.02	0
171285	1	12.16	128.47	68.97	1273.04	65.82
171287	1	45.8	0	7.44	336.86	0
171288	1	211.44	0	0	577.63	0
171290	1	208.42	144.13	72.65	1095.82	52.18
171291	1	0	0	38.04	1020.19	0
171292	1	0	60.51	0	871.98	0
171294	1	0	0	0	720.6	0
171296	1	0	137.48	0	1022.29	62.69
171303	1	79.5	28.3	70.07	1619.66	64.43
171313	1	0	0	0	284.28	0
171316	1	29.89	172.89	0	1270.73	65.89
171319	1	123.99	0	29.74	1556.96	43.93
171339	1	95.64	0	0	1873.42	0
171342	1	130.59	182.97	0	503.2	21.27

171344	1	90.76	95.81	0	520.79	20.7
171345	1	215.42	89.96	0	1441.07	74.12
171348	1	0	0	0	262.02	0
171354	1	15.08	35.3	0	699.7	0
171355	1	0	0	0	524.43	0
171373	1	0	0	0	208.05	0
171375	1	0	0	0	584.06	0
171396	1	95.65	47.96	41.45	733.61	11.67
171397	1	0	0	17.69	374.94	0
171402	1	22.32	0	0	184.99	0
171413	1	136.85	0	0	640.35	0
171439	1	0	19.12	0	321.58	0
171440	1	0	24.84	0	268.45	0
171441	1	0	0	0	66.1	0
171442	1	0	0	0	253.81	0
171445	1	0	0	0	644.06	0
171446	1	157.5	145.92	194.61	1813.43	111.95
171457	1	0	0	0	139.99	0
171465	1	0	0	0	228.01	0
171466	1	0	10.92	0	309.83	0
171469	1	0	0	0	244.21	0
171473	1	0	0	0	56.18	0
171474	1	0	0	0	254.09	0
171515	1	138.1	0	0	1133.84	0
171516	1	40.87	0	0	393.9	0
171518	1	0	0	0	901.24	0
171519	1	0	0	0	101.36	0
171527	1	223.53	0	0	1729.62	0
171533	1	0	0	0	182.38	0
171534	1	52.83	0	0	262.74	0
171540	1	97.34	70.86	76.94	881.79	0
171552	1	327.16	192.21	154.75	1380.59	103.3
171553	1	105.12	99.13	48.84	825.58	0
171554	1	105.82	92.14	39.66	769.28	0
171556	1	104.6	89.59	0	409.7	0
171563	1	0	0	0	580.58	0
171570	1	76.33	188.12	94.33	1033.46	44.05
171572	1	31.07	101.24	51.97	1266.6	20.11
171580	1	160.69	107.78	82.36	1583.62	43.15
171589	1	0	0	0	566.98	0
171591	1	195.36	155.35	140.32	1912.56	0
171597	1	0	0	11.02	1100.91	15.05
171598	1	105.05	171.36	0	1778.89	0
171601	1	98.98	29.39	58.24	938.18	0
171609	1	196.31	0	23.6	720.19	0
171707	1	0	0	0	399.14	0
171708	1	0	0	0	0	0
171730	1	34.11	0	394.2	666.92	0
171731	1	0	0	0	460.82	0
171743	1	287.78	0	0	2164.25	0

171745	1	109.12	64.83	90.52	888.63	64.76
171746	1	32.58	0	0	362.21	0
171751	1	281.64	0	113.28	1315.43	0
171785	1	539.72	469.29	161.36	3003.63	296.68
171817	1	6820.03	1918.9	0	1171.25	0
171825	1	0	0	0	356.43	0
171831	1	178.06	0	34.05	576.02	0
171835	1	0	170.57	64.65	629.06	0
171840	1	266.71	0	0	593.85	0
171851	1	0	191.86	33.73	1282.11	0
171852	1	159.96	158.42	117.41	1106.82	0
171866	1	0	104.74	0	795	0
171873	1	388.42	0	0	1057.09	0
171892	1	0	91.36	0	576.98	0
171893	1	147.75	79.06	0	833.26	0
171896	1	116.94	77.6	0	316.8	12.93
171897	1	73.2	0	0	322.81	0
171899	1	11.28	0	0	208.81	0
171900	1	0	0	0	590.88	0
171903	1	59.52	50.29	0	402.59	0
171905	1	245.53	46.43	0	350.9	0
171920	1	396.48	532.66	144.41	2105.24	153.06
171923	1	135.69	132.53	93.1	816.28	0
172094	1	0	0	0	0	0
182931	1	84.57	25.95	0	1079.43	0
182960	1	0	0	0	511.21	0
192465	1	0	0	0	150.99	0
192471	1	0	76.06	0	381.01	0
192472	1	0	0	66.73	954.63	0
193564	1	0	0	14.97	481.69	0
193574	1	0	0	0	297.13	0
193585	1	0	0	0	263.6	0
193589	1	0	0	0	55.9	0
193599	1	0	0	0	237.45	0
193601	1	0	0	0	1518.35	0
193604	1	132.33	0	0	980.66	0
195802	1	224.49	121.17	0	307.75	0
195803	1	488.85	0	35.93	979.29	75.27
195819	1	124.78	97.82	78.94	1189.97	88.55
195822	1	150.05	87.82	68.11	1027.34	110.11
195834	1	98.42	90.26	60.57	1034.62	44.6
195858	1	0	0	0	986.81	0
195894	1	0	0	56.63	928.71	39.7
195912	1	61.39	0	98.44	1965.33	36.43
195916	1	213.92	155.29	149.67	1126.79	93.93
195922	1	382.03	313.61	253.51	1999.34	171.24
195930	1	117.79	67.52	44	1352.5	45.68
195961	1	98.74	148.87	0	1121.19	95.27
195973	1	36.02	29.22	0	319.79	20.22
195976	1	161.06	0	0	749.81	0

195983	1	285.46	158.09	133.09	1268.56	68.6
196010	1	0	66.57	60.49	1274.3	115.67
196016	1	71.01	72.76	24.44	571.52	19.44
196030	1	90.15	0	60.96	694.02	0
196052	1	97.7	79.49	143.87	1301.12	79.85
196057	1	299.26	196.21	138.08	1203.47	61.67
196076	1	170.67	125.78	117.03	1118.35	44.26
196110	1	122.61	13.47	0	1438.56	0
196111	1	443.26	347.06	378.85	3137.53	349.66
196126	1	155.12	160.83	162.73	1270.89	121.13
196133	1	0	135.8	0	456.55	0
196527	1	45.22	48.78	60.2	561.3	31.79
196532	1	0	0	0	0	0
196557	1	561.31	0	305.16	651.7	0
196577	1	169.12	0	0	759.92	0
196640	1	355.14	0	15.8	1201.02	69.42
196805	1	262.95	289.73	188.23	2303.32	62.11
196806	1	254.03	227.62	123.28	1737.78	108.25
196811	1	258.2	220.67	123.82	1280.02	162.37
197851	1	0	301.33	0	1233.86	0
197852	1	137.48	247.78	42.19	1445.99	26.9
197862	1	331.07	221.01	105.46	1330.61	52.24
197905	1	86.11	0	0	672.13	0
197931	1	125.08	79.31	41.55	1374.65	23.25
197943	1	82.95	0	63.28	1092.41	29.03
197944	1	176.46	0	42.2	716.15	51.09
198018	1	0	0	34.96	1142.54	0
198021	1	38.68	0	0	1141.58	0
198023	1	136.48	119.06	55.02	1220.18	70.71
199182	1	48.25	57.98	72.22	1033.74	53.4
199205	1	153.12	313.14	421.15	2595.46	132.92
200195	1	158.67	216.01	328.33	2686.87	328.78
200210	1	258.72	0	396.73	1455	99.12
202858	1	254.03	138.25	58.6	745.58	62.19
202864	1	225.42	0	1503.82	1530.05	0
204121	1	181.49	0	88.23	1527.67	0
204136	1	31.74	0	0	527.42	0
204140	1	313.26	0	0	401.92	0
204143	1	0	167.19	128.23	1283.7	173.49
204146	1	0	0	0	1428.84	0
204147	1	241.45	54.86	0	1633.65	0
204160	1	0	0	0	0	0
204161	1	0	0	0	482.69	0
204163	1	116.01	146.63	0	1149.23	50.2
213707	1	0	0	0	172.16	0
213711	1	142.26	124.27	154.58	1079.71	62.7
213734	1	78.25	96	133.06	1055.02	60.68
213740	1	36.37	0	0	310.03	0
213750	1	210.45	0	176.42	1030.8	0
213755	1	303.82	296.11	78.04	1670.38	0

213760	1	0	0	0	238.61	0
213765	1	762.83	0	0	909.48	0
213770	1	21.77	0	0	449.8	0
213792	1	265.94	0	0	831.17	0
213799	1	0	0	0	117.74	0
213808	1	0	0	0	267.46	0
213892	1	130.77	0	0	579.56	0
213896	1	134.28	0	23.49	527.58	0
213906	1	129.32	0	62.14	304.96	0
213917	1	211.52	0	66.48	657.89	0
213919	1	210.54	0	0	861.24	0
213934	1	104.19	23.03	35.17	594.7	0
213948	1	234.66	107.26	182.68	1493.4	121.74
213951	1	135.29	0	33.87	848.51	38.54
213953	1	69.24	16.71	19.73	253.53	0
213956	1	117.52	86.53	82.16	739.76	0
213982	1	242.65	123.02	151.78	1146.39	77.75
213989	1	149.84	0	5	472.72	0
214000	1	417.24	299.47	0	0	0
214006	1	43	22.58	0	431.19	13.05
214013	1	343.02	178.32	163.89	2031.44	198.61
214014	1	652.18	704.95	160.88	888	0
214026	1	143.7	234.99	0	701.49	0
214029	1	11.86	14.6	0	155.81	0
214031	1	0	76.17	0	223.56	0
214035	1	35.33	78.98	0	344.03	0
214039	1	233.49	176.43	51.72	1360.98	107.61
214040	1	0	0	0	428.88	0
