# Supplementary material for: Association of Urinary Collagen Type III Degradation Product With Kidney Function and Fibrosis in Chronic Kidney Disease Patients
Source: Proteomics. 2025 Apr 10;25(11-12):e202400354. doi: 10.1002/pmic.202400354 (PMC12205290; doi:10.1002/pmic.202400354)
Supplement: Supplementary file 3 — Supporting Information [file PMIC-25-e202400354-s001.pdf]

Table S2B: Normalized peptide intensities per urine sample (analysis ID) in the dataset with IFTA recorded.

Analysis ID	e13566	e13662	e14644	e14735	e14821
203894	203.98	0	18.22	163.96	0
203896	184.53	0	0	408.61	0
203898	0	0	0	153.11	0
203899	66.26	43.66	0	414.33	0
203900	0	0	63.29	1217.3	0
203901	497.23	295.11	115.68	1457.52	65.9
203902	409.32	330.2	259.28	1176.32	0
203903	241.71	0	21.73	446.02	12.35
203904	40.42	0	0	172.55	0
203905	181.26	0	48.96	351.79	0
203906	308.88	228.64	306.62	1106.69	0
203907	0	0	0	172.34	0
203908	574.35	53.5	0	2991.8	0
203909	200.56	0	39.33	352	0
203910	547.32	289.58	497.07	2553.83	255.58
203911	172.15	0	80.66	362.7	0
203912	471.27	178.18	366.15	1344.16	0
203913	176.31	122.4	51.29	851.59	0
203914	39.67	0	0	349.26	0
203915	1000.07	131.29	203.94	1034.84	0
203916	0	0	0	280.9	0
203917	402.26	208.82	0	1307.33	75.63
203919	264.95	0	70.45	926.99	0
203920	344.74	0	66.03	557.96	0
203922	0	0	0	535.86	0
203924	65.2	0	7.92	156.87	0
203925	913.08	0	0	1017.03	65.69
203928	137.86	0	0	218.06	0
203929	80.02	172.77	0	532.14	0
203930	466.62	191.92	0	1305.13	43.31
203933	24.37	0	19.8	518.13	0
203934	0	0	0	685.25	0
203935	676.82	70.82	0	771.51	26.12
203936	423.76	40.15	556.97	1461.81	0
203937	58.04	0	43.54	603.93	0
203938	64.57	165.62	0	1438.15	189.59
203940	20.18	0	19.93	365.47	0
203941	412.65	162.53	272.3	1459.05	92.69
203943	462.84	374.72	441.11	1559.73	0
203944	172.52	231.15	105.67	1128.47	158.69
203952	61.68	23.68	42.03	219.07	0
203953	245.58	99.86	0	1663.49	111.16
203955	506.81	434.25	364.74	2593.39	262.8
203956	0	0	0	0	0
203957	0	0	0	440.66	0
203960	0	121.05	0	139.44	0
203963	308.85	101.76	0	418.33	0

203976	202.54	189.23	0	981.54	0
203977	1541.09	151.02	0	265.08	0
203978	109.46	0	0	241.88	0
203979	0	70.83	42.23	1064.67	0
203980	410.78	371.37	0	2387.19	258.96
203981	324.01	229.35	0	746.66	0
203982	420.48	313.66	115.14	1155.58	29.67
203983	127.92	95.17	40.47	219.71	0
203985	401.15	21.74	0	691.86	0
203986	511.87	0	18.35	451.1	0
203987	247.27	197.74	0	1060.87	30.62
203991	889.77	0	0	475.91	0
203992	56.51	0	0	210.78	0
203993	330.9	104.2	0	0	0
203994	0	0	0	885.14	0
203995	0	0	0	0	0
203996	56.06	40.16	0	250.39	0
203997	264.6	153.9	248.08	1372.93	38.61
203998	426.53	0	87.49	1093.27	0
204029	61.17	0	0	314.78	0
204031	16.16	46.65	0	220	0
204033	318.14	191.59	138.25	914.98	86.36
204037	72.44	191.65	0	940.67	186.11
204039	203	0	34.73	164.82	0
204043	454.21	0	363.64	1008.02	0
204046	0	0	18.44	306.62	0
204049	115.57	104.69	94.15	909.25	0
204052	94.36	0	27.76	553.85	0
204053	589.97	271.37	158.37	877.88	58.28
204057	75.16	38.47	35.73	196.65	31.26
204402	0	0	0	199.04	0
204408	0	148.09	0	0	0
204409	0	0	0	191.84	0
206473	137.41	0	0	81.05	0
206476	59.1	62.39	31.61	263.22	0
206478	154.87	0	0	106.55	0
206480	251.59	0	0	319.67	0
206484	0	0	0	0	0
206485	177.48	313.26	780.77	6152.82	483.21
206487	419.62	308.56	339.19	1676.23	167.26
206490	0	0	0	0	0
206493	973.85	0	0	495.25	0
206494	162.91	0	0	212.25	0
206496	136.18	175.02	98.82	1333.43	105.35
206499	349.54	0	0	417.79	0
206500	442.39	251.59	73.56	833.68	0
206502	312.1	0	792.15	1205.79	0
206508	192.47	0	432.08	624.56	0
206517	373.05	0	0	596.69	0
206520	299.98	0	0	54.05	0

206523	66.58	261.67	0	1529.51	0
206528	332.31	0	0	611.06	0
206530	0	0	0	0	0
206535	116.24	72.33	50.59	721.52	0
206541	91.61	173.44	0	528.28	76.77
206546	314.68	0	0	452.14	0
206550	459.28	0	0	664.42	0
206557	224.17	324.83	0	2029.25	273.49
206560	257.02	0	0	447.28	0
206563	324.11	0	223.28	963.93	0
206565	569.64	488.76	423.94	3392.81	316.71
206566	393.19	246	407.53	2340.94	204
206569	799.13	1291.61	0	344.57	0
206570	537.58	0	239.51	0	68.82
206572	0	107.76	60.72	1566.8	0
206573	0	0	0	318.17	0
206574	0	0	0	427.6	0
206579	0	0	0	245.72	0
206581	229.91	0	0	922.77	0
206582	268.24	0	0	471.83	0
206583	350.94	489.91	297.81	2018.93	0
206584	356.5	0	0	558.54	0
206585	379.41	182.92	0	1017.35	0
206586	129.82	283.86	0	1043.6	0
206590	0	0	0	286.62	0
206595	0	0	0	0	0
206600	0	0	0	0	0
206601	425.14	0	0	1121.14	0
206621	86.63	0	0	154.22	0
206624	0	0	0	0	0
206626	0	0	0	0	0
206627	0	0	0	714.36	0
206628	444.68	0	116.88	541.17	0
206630	0	0	0	383.72	0
206631	84.19	0	0	83.82	0
206632	425.22	203.51	0	1431.78	0
206633	0	156.66	0	767.82	0
206634	0	0	0	460.76	0
206635	0	0	0	578.34	0
206636	0	96.98	0	397.2	0
206637	115.57	112.77	197.83	1960.63	54.88
206638	97.14	0	0	121.51	0
206639	0	0	0	0	0
206640	226.97	171.73	0	1318.54	0
206641	0	111.86	122.74	456.39	0
206642	144.1	0	0	244.91	0
206643	395.57	299.08	0	1282.9	60.92
206644	420.12	351	0	1998.93	73.9
206647	829.29	0	36.21	1218.38	0
206648	0	0	0	899.19	0

206649	341.26	141.35	0	787.92	22.81
206650	87.33	0	21.04	545.37	0
206652	278.84	320.33	0	1766.18	162.4
206653	0	0	0	0	0
206654	0	0	0	409.77	0
206664	0	0	0	562.47	0
206665	132.62	0	10.43	506.97	8.7
206670	0	0	77.06	78.64	0
206672	0	0	0	63.53	0
206677	0	0	0	331.85	0
206678	174.83	173.48	0	1187.99	0
206679	105	76.97	15.3	600.6	0
206682	696.86	259.33	635.52	1230.69	0
206684	249.4	321.16	0	1682.86	162.59
206685	0	0	0	211.06	0
206689	0	0	0	336.78	0
206690	363.95	204.31	0	1763.06	200.99
206691	0	0	0	468.7	0
206692	0	0	0	548.05	0
206693	280.8	0	0	415.05	0
206699	343.61	302.65	256.16	1706.51	142.54
206700	210.93	0	0	183.36	0
206703	0	0	0	302.89	0
206704	0	0	0	463.83	0
206705	1814.98	79.56	0	820.78	0
206706	370.77	0	0	170.86	0
206709	89.53	0	0	723.99	0
206711	196.75	0	0	222.92	0
206713	136.25	136.83	0	829.47	25.41
206717	0	0	0	649.49	0
206718	196.88	0	0	339.87	0
206719	237.97	0	164.57	1217.76	30.15
206720	273.68	0	0	1165.32	0
206722	0	0	0	1098.06	0
206723	470.75	0	0	1236.08	0
206725	0	0	0	0	0
206726	0	0	0	994.6	0
206727	99.9	0	0	968.71	32.31
206728	723.83	41.43	56.85	411.89	0
206730	193.96	151.23	0	252.29	18.13
206731	0	0	0	740.5	0
206734	253.13	0	0	676.28	0
206736	325.42	306.86	196.36	1406.59	44.92
206738	186.75	0	0	852.19	0
206740	0	0	246.07	2528.4	0
206742	134.49	0	0	737.33	0
206743	0	0	0	276.19	0
206744	463.35	428.08	0	3383.37	418.35
206745	0	0	0	467.1	0
206746	211.93	144.88	76.47	502.74	0

206748	229.27	0	110.48	378.94	0
206749	344.37	373.35	160.13	2070.13	139.51
206750	40.27	0	0	97.85	0
206751	0	0	0	660.72	0
206755	0	0	0	340.7	0
206756	116.72	0	0	257.76	0
206757	0	0	0	437.61	0
206759	1418.11	376.85	0	906.95	0
206760	1074.58	0	397.52	1118.74	0
206761	236.92	0	0	348.86	30.17
206765	319.75	0	0	313.74	0
206766	0	0	0	1605.71	0
206767	515.24	496.62	373.11	1281.22	0
206768	465.41	0	0	738.39	29.06
206772	216.34	135.73	472.24	1182.72	0
206773	0	0	0	119.65	0
206774	241.72	232.6	200.61	970.57	0
206777	218.82	0	0	352.1	0
206781	346.71	0	361.9	2011.02	62.95
206782	287.82	123.87	46.56	978.97	0
206784	829.85	0	1187.57	2464.87	0
206790	355.39	357.52	88.36	1136.35	0
206802	97.35	0	52.86	429.8	0
206803	450.67	324.21	0	2140.9	77.13
206806	288.72	0	0	728.59	20.78
206807	386.17	0	153.81	1260.73	46.37
206808	0	53.15	0	143.46	0
206809	0	0	0	661.52	0
206810	0	125.65	0	165.76	0
206811	0	0	0	0	0
206812	0	0	0	1139.56	193.6
206813	761.13	0	306.5	1433.77	0
206816	25.89	0	0	298.87	0
206817	539.48	0	0	0	0
206821	312.75	0	127.44	723.97	0
206825	168.82	104.09	171.27	1272	0
206827	0	0	0	0	0
206828	0	0	0	182.91	0
206829	54.47	238.7	0	404.95	0
206830	381	238.49	0	1525.5	16.14
206831	277.05	0	151.45	870.31	60.04
206832	49.45	100.66	0	476.13	0
206838	671.19	0	0	2034.46	0
206839	683.82	0	0	0	0
206841	361.07	254.84	252.04	2103.85	108.42
206842	0	0	47.08	629.69	0
206844	511.48	0	4431.62	789.94	0
206847	0	162.11	40.52	179.7	0
206856	236.05	0	129.36	466.01	0
206860	230.32	196.32	0	891.14	143.76

206862	0	0	0	621.77	0
206863	679.8	374.79	0	2270.42	144.18
206865	171.99	0	0	217.02	0
206866	472.67	0	0	0	0
206867	119.81	0	19.48	194.33	0
206868	77.71	182.93	0	1054.45	0
206869	1036.76	0	0	319.13	264.72
206870	21.54	203.08	45.6	573.81	0
206872	265.29	207	78.34	438.52	0
206873	78.97	0	10.95	503.63	0
206874	199.97	0	0	0	0
206879	479.06	0	0	442.71	0
206880	1271.6	0	0	813.67	0
206885	177.37	0	29.03	340.83	0
206886	305.1	0	0	180.77	0
206887	397.19	160.28	53.91	939.94	0
206888	139.58	171.33	50.58	632.1	0
206889	33.35	120.54	89.04	770.03	27.43
206891	112.1	225.04	102.6	1076.1	34.24
206895	224.51	0	0	49.02	0
206897	92.71	0	0	287.23	0
206900	163.63	172.97	0	513.39	0
206904	0	0	0	720.39	0
206906	142.99	158.23	283.47	1406.52	107.96
206912	451.73	463.21	442.04	2713.21	273.03
206918	184.45	0	0	140.14	0
206921	203.9	0	49.61	598.89	0
206928	175.61	124.75	39.54	1007.32	125.3
206930	658	0	0	770.32	0
206931	46.92	0	0	382.18	0
206941	274	68.44	152.34	489.07	0
206943	298.91	0	0	560.11	0
206944	135.4	74.03	83.72	972.67	90.04
206945	590.96	563.98	490.92	3287.61	252.03
206952	309.03	0	0	377.74	0
206953	462.94	566.52	315.44	2193.23	281.38
206957	253.17	376.65	327.12	2654.62	0
206962	178.9	198.13	208.55	2779.4	162.89
206963	298.99	0	0	181.46	0
206964	151.12	175.5	66.86	1167.55	23.56
206965	187.24	64.94	74.51	1147.9	97.27
206966	79.95	108.38	0	711.16	15.1
206967	0	200.33	0	1223.89	0
206968	95.13	98.93	0	468.09	0
206972	0	0	130.92	1994.7	42.2
206973	0	0	0	0	0
206974	21.96	279.52	92.07	1088.13	0
206975	173.55	0	0	1235.69	35.72
206976	940.13	0	0	2661.83	0
206979	0	0	0	471.82	0

206981	122.83	0	0	464.42	0
206986	216.03	317.79	69.73	1521.2	0
206988	0	76.26	0	670.92	0
206989	534.3	148.4	0	995.6	0
206993	0	138.21	0	681.54	0
206994	549.59	567.34	442.16	3350.41	244.41
206995	260.67	0	0	374.1	0
206996	0	0	0	0	0
206997	483.13	0	0	0	0
206998	276.88	297.12	290.79	2402.51	101.08
206999	742.36	199.49	397.77	448.54	0
207000	324.69	402.57	342.21	1895.49	133.57
207001	772.92	0	0	554.95	237.64
207003	507.58	0	0	705.98	0
207004	0	0	0	168.42	0
207005	248.05	0	0	814.82	0
207006	213.71	103.02	13.74	382.42	0
207007	235.3	145.97	89.84	877.23	0
207010	236.51	0	222.73	801.09	0
207012	131.24	0	0	1431.24	191.11
207013	185.49	140.85	130	501.33	0
207014	0	0	0	1125.4	0
207019	381.57	202.34	364.4	1169.82	96.23
207020	0	87.27	0	574.67	0
207021	111.52	0	0	0	0
207022	125.67	0	28.3	358.88	0
207023	0	0	86.45	1136.06	49.34
207024	54.31	146.28	0	677.4	0
207025	1013.59	0	0	591.93	0
207026	116.98	0	0	669.03	0
207027	30.25	0	0	45.52	0
207028	78.29	77.45	40.12	616.65	0
207029	132.08	0	0	752.87	0
207033	0	275.45	0	293.84	0
207036	61.21	0	0	72.61	0
207037	142.11	203.28	74.83	1132.79	0
207039	484.83	398.84	306.31	2085.26	168.77
207041	275.44	0	64.84	920.23	256.92
207042	155.82	0	49.98	716.09	0
207047	708.67	0	0	0	0
207048	242.92	116.15	0	742.9	0
207049	578.35	0	0	204.64	0
207050	233.74	587.38	0	2850.65	214.12
207051	0	0	0	0	0
207052	0	176.19	0	415.09	0
207615	1934.64	0	156.32	0	0
207617	681.34	0	0	0	0
207618	149.41	169.65	0	524.14	0
207620	564.48	0	0	1445.04	241.98
207621	198.18	109.23	183.45	669.59	48.43

207622	539.06	0	40.59	1675.71	0
207624	1167.28	0	458.19	885.79	165.91
207625	533.34	143.47	0	1232.04	60.18
207626	1052.31	0	1927.85	550.32	500.37
207628	153.9	143.93	86.24	1618.8	119.89
207629	232.12	383.98	0	2124.9	133.78
207630	179.03	0	0	421.56	0
207631	154.94	127.57	0	1108.96	68.43
207634	340.58	0	469.06	1730.47	0
207639	0	0	127.19	0	0
207642	281.34	173.23	2381.44	584.1	0
207649	259.51	513.14	0	0	0
207650	0	0	0	366.52	0
207651	0	0	0	211.39	0
207654	345.67	0	78.76	490.34	0
207657	894.1	0	209.85	767.5	82.4
207658	42.39	129.03	0	814.44	0
207660	685.71	570.63	680.6	1240.56	0
207661	23.75	0	40.06	823.41	0
207662	0	0	0	625.83	0
207663	231.92	0	0	118.47	0
207664	0	0	0	345	0
207665	0	104.51	0	418.78	0
207666	225.64	262.41	243.32	1501.01	163.12
207668	0	0	0	649.57	0
207669	385.07	0	0	234.97	0
207670	1315.88	0	0	166.88	0
207671	1371.09	0	0	0	0
207685	4806.67	0	0	0	0
207720	7298.46	0	0	0	0
207739	1007.25	0	0	343.3	0
207745	1668.71	0	1707.43	0	0
207748	38.42	0	0	383.19	0
207749	135.42	146.09	0	936.89	18.95
207750	513.39	0	0	712.92	0
207751	0	0	0	32.84	0
207752	14.97	0	0	424.92	27.08
207753	0	50.11	0	692.16	0
207755	98.32	0	49.48	725.97	0
207756	320.1	233.06	0	1472.89	160.62
207757	416.16	138.95	139.87	588.87	0
207758	0	0	0	334.6	0
207759	107.3	40.49	0	555.4	21.87
207760	0	0	0	1063.51	34.15
207761	0	0	0	1022.49	0
207762	0	0	64.16	1256.72	0
207763	0	0	0	164.81	0
207764	0	0	0	0	0
207765	0	112.24	0	737.38	0
207766	105.89	0	0	517.07	0

207767	548.04	269.17	240.58	1050.94	0
207768	0	0	0	120.2	0
207769	616.69	165.07	0	605.63	0
207770	197.68	0	271.02	2004.98	150.13
207771	148.51	192.19	0	923.03	0
207772	711.77	0	0	397.35	0
207773	0	75.92	0	50.75	0
207774	408.81	0	0	793.4	0
207775	1129.78	0	2962.31	2771.98	0
207776	0	0	0	389.38	0
207777	144.08	0	116.52	538.1	0
207778	37.09	0	0	371.44	0
207780	0	0	0	219.56	0
207781	0	0	0	0	0
207782	20.24	132.65	0	898.82	35.96
207783	164.07	79.17	193.33	754.86	41.63
207784	0	0	0	753.12	0
207785	0	0	0	0	0
207786	0	232.91	0	170.85	0
207787	0	0	0	370.2	0
207788	206.84	115.59	148.85	591.15	20.62
207789	0	52.82	0	124.02	0
207791	494.62	0	0	733.43	0
207792	1458.5	0	5160.3	2110.17	342.8
207797	397.95	0	495.08	319.8	0
207804	116.64	0	79.34	594.88	0
207808	420.07	0	0	209.19	0
210534	2596.22	0	0	363.48	0
210536	1100.3	61.02	0	590.66	0
210538	465.62	73.33	0	825.54	170
210541	1026.81	2299.27	0	1092.93	0
210542	0	0	0	0	0
210544	9.51	0	0	0	0
210545	667.94	0	0	0	0
210551	370.9	0	68.23	86.61	68.86
210552	0	0	0	0	0
210556	185.06	106.41	169.33	898.39	37.44
210558	946.7	2779.05	6403.56	967.09	282.32
210560	0	0	0	0	0
210564	475.35	98.05	84.4	502.35	0
210565	424.94	128.13	976.6	516.22	0
210566	5129.25	0	0	0	0
210568	0	0	0	173.1	0
210569	1308.12	0	0	128.08	0
211041	0	0	0	91.48	0
211044	1460.13	0	0	0	0
211049	0	0	0	0	0
211050	707.45	0	0	0	0
211051	57.31	0	0	0	0
211052	3967.18	0	0	431.15	31.32

211056	551.54	0	0	209.66	0
211057	0	0	0	377.77	0
211062	759.97	609.99	0	0	0
211064	0	0	0	0	0
211065	121.55	0	0	154.34	0
211066	0	0	0	159.58	0
211067	867.84	0	0	1588.18	0
211068	0	0	0	288.61	0
211069	185.95	0	0	58.39	0
211071	5226	185.64	0	536.72	0
211073	0	0	0	0	0
211075	0	0	0	78.85	0
211080	0	0	0	73.33	0
214723	4743.72	0	0	0	0
221269	297.47	0	0	516.44	0
221270	0	0	0	632.74	0
221271	75.15	0	0	387.81	0
225464	0	0	493.36	1358.39	0
225467	1404.44	0	0	1216.12	0
